# Increased Risk of Multiple Outpatient Surgeries in African-American Carriers of *Transthyretin* Val122Ile Mutation Is Modulated by Non-Coding Variants

**DOI:** 10.3390/jcm8020269

**Published:** 2019-02-22

**Authors:** Renato Polimanti, Yaira Z. Nuñez, Joel Gelernter

**Affiliations:** 1 Department of Psychiatry, Yale University School of Medicine and VA CT Healthcare Center, West Haven, CT 06516, USA; yari.nunez@yale.edu (Y.Z.N.); joel.gelernter@yale.edu (J.G.); 2 Departments of Genetics and Neuroscience, Yale University School of Medicine, New Haven, CT 06510, USA

**Keywords:** TTR amyloidosis, African ancestry, phenome-wide, V122I, medical history

## Abstract

**Background:** African-Americans (AAs) have a 3.5% carrier prevalence of *Transthyretin* (*TTR*) Val122Ile mutation (rs76992529), which is the genetic cause of a hereditary form of amyloidosis. **Methods**: We investigated the medical history of Val122Ile carriers and assessed the role of a non-coding variation in 4361 unrelated AAs. **Results**: We observed that the Ile122 allele was associated with a 6.8-fold increase in the odds of having 10 or more outpatient surgeries (*p* = 7.81 × 10^−5^). Stratifying the analysis by sex, the Ile122 allele was associated with a 15.2-fold increase in the odds of having 10 or more outpatient surgeries in men (*p* = 6.49 × 10^−7^). A similar sex difference was observed with respect to the association of Val122Ile with musculoskeletal and connective-tissue disorders in an independent cohort of British subjects (*n* = 361,194, *p* = 2.47 × 10^−13^; *n*_male_ = 167,020, *p*_male_ = 4.02 × 10^−24^). In Val122Ile African-American carriers, we observed that haplotypes in the upstream region regulating *TTR* hepatic expression are associated with having 10 or more outpatient surgeries (*p* = 2.56 × 10^−9^). **Conclusions**: *TTR* Val122Ile showed a large effect with respect to an extreme phenotype identified in medical history that may be related to osteoarthritis, an early sign of the disease. Additionally, the non-coding variation appears to accelerate the negative consequences associated with Val122Ile mutation via *TTR* expression regulation.

## 1. Introduction

Transthyretin (TTR) amyloidosis is a life-threatening disease (Online Mendelian Inheritance in Man: #105210) caused by coding mutations in the *TTR* gene. The disease is characterized by a wide range of clinical signs and symptoms, including peripheral neuropathy (sensory and motor), cardiomyopathy, autonomic neuropathy, gastrointestinal impairment, nephropathy, and ocular deposition [1,2]. Although more than 100 amyloidogenic variants have been identified in *TTR*, two mutations account for most of the cases worldwide, i.e., Val30Met (rs28933979) and Val122Ile (rs76992529) [3,4]. While Val30Met is mainly present in European and Japanese populations [5], V122Ile shows high frequency in individuals of African descent. In African Americans (AAs), the Ile122 allele frequency is 1.5%, which corresponds to 3.5% of carriers [6,7]. The main disease phenotype associated with this mutation is restrictive amyloid cardiomyopathy, and it has been reported that homozygous individuals can be symptomatic in their late 50s and early 60s, while heterozygotes can develop congestive heart failure between the ages of 60 and 80 [8]. However, the actual extent of Val122Ile penetrance is still debated because of the differences observed between general-population cohorts and case–control studies [4]. Penetrance variability has also been observed for other *TTR* mutations, and previous studies suggested that genetic and environmental factors play a role in the phenotypic expression [9,10,11,12]. In particular, recent investigations observed that non-coding variation of the *TTR* gene may play a role in the complex genotype–phenotype correlation due to its effect on gene expression regulation [13,14]. Although no analysis has been conducted previously to test this hypothesis on Val122Ile AA carriers, an *in silico* study based on the 1000 Genomes Project reference panel observed that some Ile122 haplotypes present different non-coding structures, including multiple regulatory variants [10]. The observation that the known risk alleles occur on different haplotypic backgrounds with different regulatory variants supports the claim that the non-coding variation may modulate the phenotypic presentation among Val122Ile carriers.

The aim of the present study was to evaluate two hypotheses: (i) Val122Ile affects the medical history of carriers earlier than previously reported; (ii) Non-coding regulatory variation modulates the effects of Val122Ile among carriers. To test these hypotheses, we investigated a relatively young cohort (interquartile age range of 35 to 48 years) including 4361 unrelated AAs with 152 Val122Ile carriers (150 Val122/Ile122 heterozygotes and two Ile122/Ile122 homozygotes). The initial analysis tested whether there were differences in the medical history (health status, primary diagnoses, hospitalizations, outpatient surgeries, emergency care events, and prescribed medications) between carriers and non-carriers. Then, we tested whether the haplotypic structure around the *TTR* gene in Val122Ile carriers affects the associations identified in the initial analysis.

## 2. Methods

### 2.1. Study Population

The sample was selected from the Yale–Penn cohort, which includes more than 14,000 participants. Subjects were recruited at five US clinical sites: Yale University School of Medicine (New Haven, CT, USA), the University of Connecticut Health Center (Farmington, CT, USA), the University of Pennsylvania Perelman School of Medicine (Philadelphia, PA, USA), the Medical University of South Carolina (Charleston, SC, USA), and McLean Hospital (Belmont, MA, USA). The institutional review board at each participating site approved the Yale–Penn project, which also extends to the present study, and written informed consent was obtained from each participant. Phenotypic information was collected using the SSADDA (Semi-Structured Assessment for Drug Dependence and Alcoholism) [15,16]. Biological samples for genetic analyses were collected from each subject at the time of the assessment. Data from the Yale–Penn cohort were mainly used to conduct genetic studies of psychiatric disorders and behavioral traits [17,18]. In the present study, we investigated 4361 unrelated individuals of African descent. As previously reported [19], ancestry and relatedness were verified using genetic information. Lifetime data regarding physical health were extracted from the medical history section of the SSADDA, which includes self-reported information regarding overall health status, primary diagnoses made by a doctor, hospitalizations, outpatient surgeries, emergency room visits, and the use of prescribed medications (Table 1).

### 2.2. Genetic Data

Biological samples for DNA extraction collected at the time of the phenotypic assessment were used to conduct genome-wide array-based genotyping. The 4361 individuals included in the present analyses were genotyped with two different genome-wide arrays: 2763 using the Illumina HumanOmni1-Quad v1.0 microarray, and 1598 using the Illumina HumanCoreExome array. Details of the genotyping procedures were described previously [20]. Val122Ile (rs76992529) was included among the markers on the Illumina HumanCoreExome array. Val122Ile genotype for the subjects analyzed with the Illumina HumanOmni1-Quad v1.0 microarray was imputed using the Michigan Imputation Server [21] and the 1000 Genomes Project Phase 3 Reference Panel [22]. We obtained high-quality imputation of the Val122Ile mutation with an INFO score = 0.98. The reliability of the imputation of the Val122Ile mutation was confirmed by the fact that we observed a perfect concordance between genotype data from the Illumina HumanCoreExome array and imputation data from the Illumina HumanOmni1-Quad v1.0 microarray in 89 individuals (3 Val122Ile carriers and 86 non-carriers) genotyped with both arrays. Haplotype association analysis in Val122Ile carriers was conducted using imputed data obtained from both genome-wide arrays. We used high-quality imputed variants (INFO score > 0.95, minor allele frequency > 5%; Table S1) in a 2Mb region that included the upstream region, *TTR* coding and non-coding sequences, and the downstream region (GRCh37/hg19 chr 18: 28,171,770–30,174,635).

### 2.3. Statistical Analysis

Plink 1.9 [23] was used to implement logistic, and linear regression analyses to calculate the association between Val122Ile and phenotypic traits (binary and quantitative, respectively) considering an additive genetic model. Quantitative traits were normalized using appropriate Box-Cox power transformations before being entered into the analysis. Quantitative traits with non-normal distribution after Box-Cox transformation were converted to binary variables using different thresholds. Age, sex, and 10 ancestry principal components were included as covariates in the regression models. For Val122Ile association analysis, we applied a Bonferroni-based threshold for multiple testing correction accounting for the number of phenotypes tested (*n* = 26, *p* = 1.92 × 10^−4^). Haploview 4.2 [24] was used to conduct the haplotype association analysis considering V122I carriers only. Linkage disequilibrium (LD) blocks were defined considering the algorithm proposed by Gabriel et al. [25]. Bonferroni correction for the haplotype association analysis accounted for the number of haplotypes tested (*n* = 590, *p* = 8.47 × 10^−5^). Functional annotation analyses were conducted using Haploreg v4.1 [26] and RegulomeDB [27]. The GTEx v7 [28] data were used to investigate how non-coding variation affects *TTR* gene expression in the liver, which is the main source of the TTR protein.

## 3. Results

**Figure 1:**
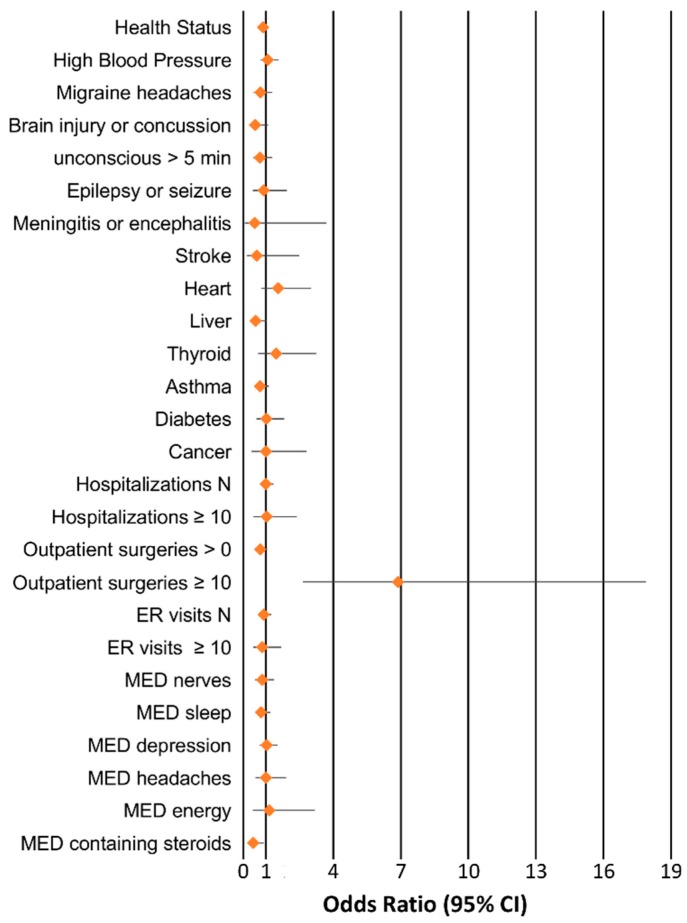
Associations between *Transthyretin* (*TTR*) V122Ile and traits related to medical history. Odds ratio and 95% confidence intervals are reported for each association.

Table 1 reports the characteristics of the sample investigated, including information regarding demographics, Val122Ile frequency, and the traits investigated in the association analysis. Among 4361 individuals, we identified 152 Val122Ile carriers (3.48%) with 150 Val122/Ile122 heterozygotes and 2 Ile122/Ile122 homozygotes. No Val122Ile carriers were identified in the Yale–Penn participants of European descent. Figure 1 shows the results of Val122Ile association analysis with respect to the tested traits related to medical history. Details of the association results are reported in Table S2.

**Figure 2:**
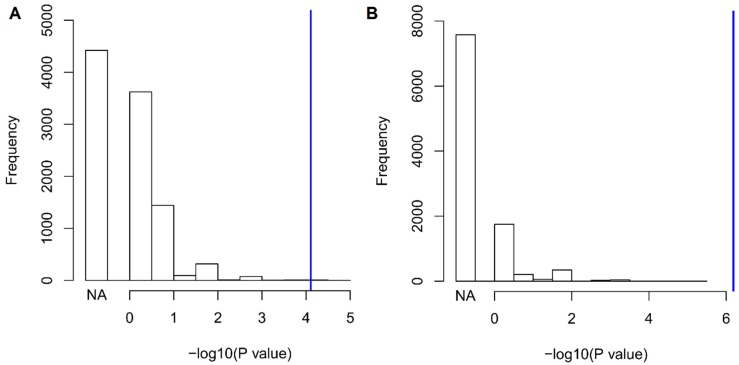
Null distribution of association results generated through 10,000 random permutations of having 10 or more outpatient surgeries with respect to Val122Ile genotype status in the whole cohort (**A**) and in the male-only sample (**B**). Blue lines indicate the observed values. The “NA” column represents the permutation results where no case is a carrier of the Val122Ile mutation.

We observed a significant association of Val122Ile that survived Bonferroni multiple testing correction: the I122 allele was associated with a 6.9-fold increase in the odds of having 10 or more outpatient surgeries, Odds Ratio (OR) = 6.87, *p* = 7.81 × 10^−5^ (2.6% carriers versus 0.5% non-carriers). Since there is previous evidence of high penetrance of the Val122Ile mutation in male carriers [29], we conducted a sex-stratified analysis for the phenotype identified. In male subjects (carriers *n* = 80, non-carriers *n* = 2357), the I122 allele was associated with a 15.2-fold increase in the odds of having 10 or more outpatient surgeries (*p* = 6.49 × 10^−7^, 5.1% carriers versus 0.3% non-carriers). In female sample (carriers *n* = 72, non-carriers *n* = 1852), no Val122Ile carriers had more than 10 outpatient surgeries (0% carriers versus 0.6% non-carriers). No age effect was observed in genetic association in the overall cohort and in the male sample (*p* > 0.05). Since the trait identified is an extreme phenotype observed in a limited number of individuals (*n* = 24), we conducted a permutation analysis to verify whether the significant association might be inflated by the number of cases. We performed 10,000 permutations of the *TTR* Val122Ile genotypes with respect to the “having 10 or more outpatient surgeries” phenotype and verified that the finding observed was significantly different from the null distribution of the results obtained from the permuted datasets considering the whole cohort and the male-only sample (whole-cohort *p*_permutation_ = 4 × 10^−4^, male-only *p*_permutation_ < 10^−4^, Figure 2). Among the permuted results, the most likely random scenario is that no case was a carrier of the Val122Ile mutation (whole cohort 4422/10,000; male-only sample 7575/10,000). In line with its clinical significance, *TTR* Val122Ile showed a large effect with respect to this extreme phenotype.

Although the association was not significant (*p* > 0.05), Val122Ile was positively associated with heart disease (OR = 1.55), and this association was slightly stronger in men than in women (OR_men_ = 1.65; OR_women_ = 1.39). To verify whether the V122Ile mutation affects the relationship between the numbers of outpatient surgeries and the diagnosis of a heart disease, we tested the association between these health-related traits in carriers and non-carriers. Among V122Ile carriers, having 10 or more outpatient surgeries was associated with a 17.5-fold increase in the odds of having a diagnosis of heart disease (*p* = 0.022). A similar association strength was observed considering the male sample only (OR = 18, *p* = 0.027). Among non-carriers, there was no association between having 10 or more outpatient surgeries and the diagnosis of a heart disease (*p* = 0.836).

To verify whether non-coding regulatory variation modulates the association between Val122Ile and having 10 or more outpatient surgeries, we conducted a haplotype association analysis in Val122Ile carriers only. We analyzed a 2 Mb region (GRCh37/hg19 chr 18: 28,171,770–30,174,635; upstream region, *TTR* gene, and downstream region), identifying 111 LD blocks that presented a total of 590 haplotypes detected (Table S3). Five haplotypes showed positive associations (i.e., higher frequency in cases than in controls) that survived Bonferroni multiple testing correction (Table 2, *p* < 8.45 × 10^−5^). Table S4 reports the association results of all haplotypes tested.

**Figure 3:**
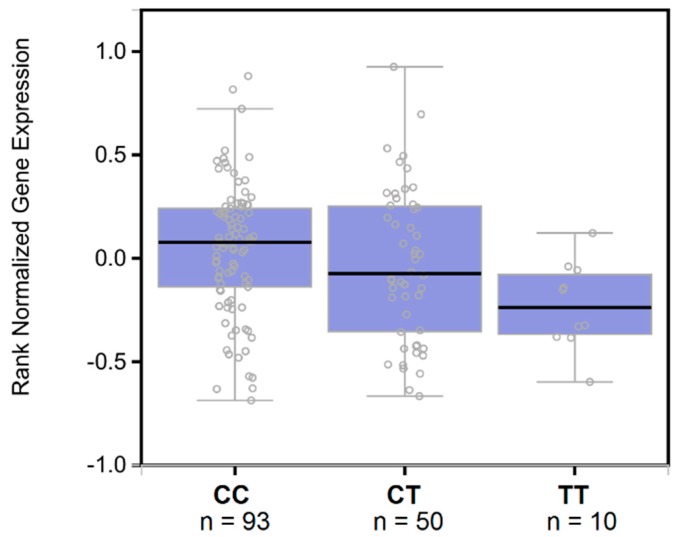
Expression quantitative trait loci box plots of associations between rs9957088 genotypes and hepatic *TTR* gene expression (data source: GTEx V7). The purple box plot shows ranked normalized gene expression in median, 1st and 3rd quartiles, and 1.5 interquartile range (IQR) of 1st and 3rd quartiles.

The significantly associated haplotypes are located in four LD blocks close to each other that cover a 253 kb region located 531 kb upstream of the *TTR* gene (Figure S1). Annotation analyses showed that variants included in these LD blocks present multiple signs of evidence of regulatory function including highly conserved sequences, enhancer histone marks, DNase hypersensitivity sites, protein binding sites, and transcription factor motif changes (Table S5). Among the variants present in the associated haplotypes, rs9957088 showed the highest functionality score (RegulomeDB score = 3a) and was significantly associated with *TTR* gene expression in the liver (C allele: beta = 0.15, *p* = 0.013, Figure 3). Additionally, we verified protein changes associated with this variant in a large cohort of healthy blood donors (*n* = 3301) [30]. Although TTR was not among the proteins tested, rs9957088 showed the strongest association with haptoglobin protein levels (C allele: beta = 0.109, *p* = 1.02 × 10^−4^), with an effect direction concordant with *TTR* gene expression.

Finally, we tested whether the effect of non-coding variation is affected by the haplotype phase of the Val122Ile mutation (cis—non-coding regulatory haplotype located on the same copy of the chromosome of Ile122 allele—versus trans—non-coding regulatory haplotype and Ile122 alleles located on different copies of the chromosome). The analysis was conducted considering the haplotype with the strongest functional evidence. We observed that the haplotype phase of the non-coding variation with respect to Val122Ile mutation did not affect the association observed (Val122 allele, *p* = 0.001; Ile122 allele, *p* = 4 × 10^−4^, Table 3).

Since no further information was available in the assessment of the Yale–Penn cohort regarding the types of outpatient surgeries, we investigated the association of the Val122Ile mutation with traits related to osteoarthritis in an independent cohort. These are recognized as early signs of the disease [31], and arthroplasty is a common procedure in outpatient settings [32]. This analysis was conducted using data of British participants from the UK Biobank. High-quality genotypic information is available for Val122Ile in the UK Biobank cohort (i.e., the variant is genotyped directly in the array used). Details regarding the data used in our analysis are available at [33]. Although there is a consistent difference in the carrier frequency of Val122Ile between our African-American cohort (3.5%) and the UK Biobank cohort (0.004%), the same clinical phenotype is present in Val122Ile carriers of European descent [34]. Accordingly, we observed a strong association of Val122Ile with the trait ICD10 M13: “Other disorders of the musculoskeletal system and connective tissue” (*n* = 361,194, beta = 0.073, *p* = 2.47 × 10^–13^). Additionally, when the analysis was stratified by sex, we observed the same male-specific associations observed in the African-American Val122Ile carriers (*n*_male_ = 167,020, beta_male_ = 0.14, *p*_male_ = 4.02 × 10^−24^, n_female_ = 194,174, beta_female_ = –0.002, *p*_female_ = 0.921).

## 4. Discussion

TTR amyloidosis is a rare life-threatening disease with complex phenotype–genotype correlations characterized by strong variability in penetrance, age of onset, and clinical symptoms [35,36]. Several studies have been conducted to understand the mechanisms determining the phenotypic variability observed among the carriers of *TTR* amyloidogenic mutations [37,38,39,40]. However, although Val30Met and Val122Ile are the most frequent *TTR* mutations, there is an evident disparity in the ongoing research efforts to understand the molecular mechanisms associated with these disease-causing variants. This is in line with the well-known population disparity in human genetic research [41]. Additionally, the generally lower socioeconomic status among the AA population compared to European Americans is a possible explanation for the general lack of representation of *TTR* Val122Ile carriers among the cohorts of patients with hereditary cardiac amyloidosis [4]. The present study aimed to reduce the population disparity in TTR amyloidosis research, investigating two aspects of *TTR* Val122Ile pathogenicity: i) The early phenotypic manifestation of the mutation in AA carriers; ii) Whether non-coding variation in regulatory elements modulates the health status of AA carriers.

The association analysis of Val122Ile with medical history identified a significant result for carriers having 10 or more outpatient surgeries. In the SSADDA assessment, outpatient surgery was defined as surgery that did not require an overnight hospital stay. Having 10 or more outpatient surgeries is an extreme phenotype that occurred in a limited number of subjects. According to our permutation analysis (*n* = 10,000), the most likely random scenario is that no Val122Ile carrier would be a case for this phenotypic trait.

Unfortunately, no further information is available regarding the type of outpatient surgeries undergone by the Yale–Penn participants. Although the main phenotypic expression of Val122Ile mutation is cardiac, multiorgan involvement was also observed among carriers, with gait, gastrointestinal, neurological, urinary/renal, and ocular symptoms also reported [36,42]. Since the age of the Yale–Penn cohort is relatively lower than the age of onset observed in patients with the *TTR* Val122Ile mutation, we hypothesize that our observed association was due to an early expression of the disease. Arthroplasty, an increasingly common procedure in outpatient settings [32], has been reported as an early sign of transthyretin cardiac amyloidosis (i.e., the main phenotype associated with *TTR* Val122Ile mutation), occurring approximately seven years before the disease diagnosis [43]. This symptom is due to the TTR amyloid deposition in the articular cartilage, contributing to the cell and extracellular matrix damage observed in osteoarthritic joints [31]. To test the hypothesis that our result regarding outpatient surgeries is related to the early occurrence of arthroplasty, we investigated the association of Val122Ile with musculoskeletal and connective-tissue disorders observed in a large independent cohort of European descent. Notwithstanding the different ancestral background, a similar clinical phenotype is expected in Val122Ile carriers of African and European descent [34]. Accordingly, we observed a strong association with musculoskeletal and connective-tissue disorders that follows the same male-specific pattern observed in the African-American cohort. Although this result supports our hypothesis, further studies are needed to confirm that outpatient surgeries in Val122Ile carriers are related to the TTR amyloid deposition in the articular cartilage.

Because of the difficulty in recognizing symptoms outside of the context of a specialized diagnostic environment, the diagnosis of TTR amyloidosis frequently occurs several years after the emergence of the first signs, with clinical procedures performed to help to alleviate the symptoms [44] (if the diagnosis is ever even made at all). Under this scenario, the association between Val122Ile and outpatient surgeries in our relatively young cohort indicates that, in certain carriers, the very early symptoms of the disease may be multiorgan rather than organ-specific. However, our data also confirmed the relationship between Val122Ile and cardiac function. We observed an effect size in the association between the *TTR* mutation and heart disease, similar to the one reported by a previous analysis of a general-population cohort [45]. Additionally, there is a relationship between having 10 or more outpatient surgeries and heart disease among the Val122Ile carriers investigated that is not present among the non-carriers. This suggests that the early signs—like the musculoskeletal and connective-tissue disorders observed in UK Biobank—causing multiple clinical procedures are linked to the cardiac symptoms.

Previous studies indicated that non-coding variation in regulatory elements is involved in the phenotypic heterogeneity observed among carriers of *TTR* mutations where penetrance, age of onset, and organ involvement are likely modulated by changes in transcriptomic regulation [13,14]. However, no previous investigation was conducted to test this hypothesis among AA carriers of *TTR* Val122Ile. In our study, we observed that, among Val122Ile carriers, genetic variability upstream of the *TTR* gene is associated with the phenotype identified in the carriers-versus-non-carriers analysis. In silico analyses confirmed that this variation is located in predicted functional elements that regulate *TTR* gene expression in the liver, the main organ source of the amyloidogenic protein. The association of Val122Ile with “having 10 or more outpatient surgeries” was not age-dependent. This suggests that certain Val122Ile carriers with a specific non-coding regulatory haplotype may show the first signs of the disease earlier than Val122Ile carriers without the regulatory non-coding haplotype. Additionally, the same *TTR* non-coding haplotypes are also associated with changes of haptoglobin protein in the blood. This result is particularly interesting since haptoglobin is one of the extracellular chaperones overrepresented in transthyretin amyloidosis [46]. Accordingly, we hypothesize that the non-coding regulatory haplotypes affect both transcriptomic and proteomic regulation of the *TTR* gene, causing the activation of extracellular chaperones to cope with amyloid-prone proteins.

## 5. Conclusions

We report novel data regarding how Val122Ile affects the health status of carriers of African descent at an earlier point in life than the expected late-onset disease. Additionally, we provide the first evidence of the role of non-coding variability and gene expression regulation in the phenotypic presentation among AA carriers, similar to what was observed in other studies for other *TTR* mutations. These data start to explain the clinical variability observed in subjects who are carriers of the disease-causing mutation. Further studies based on a deeper phenotypic characterization will be needed to explore the hypotheses generated by the present investigation. The high frequency of Val122Ile in AAs permits the investigation of a large number of individuals, providing a tool for understanding the molecular mechanisms and epidemiological associations present in TTR amyloidosis. Unlike Val122Ile allele frequency in individuals of African descent (1.5%), *TTR* amyloidogenic mutations have a lower allele frequency (<0.3%); therefore, a much larger sample size will be needed to investigate their phenotypic spectrum.

## Figures and Tables

**Table 1:** Characteristics of the study population. IQR: Interquartile range.

**Study Population (*n* = 4361)**	
**Age** years, IQR	35–48
**Sex**, Women (%)	1924 (44)
**Val122Ile**, Y (%)	152 (3.5)
**Health Status**	
Excellent (%)	675 (15)
Very Good (%)	1135 (26)
Good (%)	1420 (33)
Fair (%)	940 (22)
Poor (%)	191 (4)
**Doctor’s diagnoses**	
High blood pressure, Y (%)	1325 (30)
Migraine headaches, Y (%)	605 (14)
Brain injury or concussion, Y (%)	388 (9)
Been unconscious for longer than 5 min, Y (%)	554 (13)
Epilepsy or have had a seizure, Y (%)	213 (5)
Meningitis or encephalitis, Y (%)	57 (1)
Stroke, Y (%)	88 (2)
Heart disease, Y (%)	182 (4)
Liver disease, Y (%)	481 (11)
Thyroid disease, Y (%)	134 (3)
Asthma, Y (%)	928 (21)
Diabetes, Y (%)	374 (9)
Cancer, Y (%)	108 (2)
**Hospitalizations**	
N, IQR	0–3
≥10, Y (%)	161 (4)
**Outpatient surgeries**	
>0, Y (%)	1945 (45)
≥10, Y (%)	24 (0.6)
**Emergency room visits**	
N, IQR	0–3
≥10, Y (%)	300 (7)
**Prescription medications**	
for nerves, Y (%)	610 (14)
for sleep, Y (%)	924 (21)
for depression, Y (%)	1012 (23)
for headaches, Y (%)	292 (7)
for energy, Y (%)	95 (2)
containing steroids, Y (%)	451 (10)

**Table 2:** Haplotype associations with “having 10 or more outpatient surgeries” that survived multiple testing correction (*p* < 8.45 × 10^−5^) in the carriers-only sample (*n* = 152).

LD Block	Location (Chr18)	*TTR* Region	Haplotype	Case-Control Frequencies	Chi Square	*p* Value
#12	28,387,576-28,391,602	Upstream	GCACCGTATGGAGGGACCTC	0.375, 0.030	24.395	7.85 × 10^−7^
#14	28,456,564-28,481,528	Upstream	TCTGTGTAGGGCC	0.250, 0.007	35.494	2.56 × 10^−9^
#15	28,481,625-28,484,316	Upstream	GACAGA	0.250, 0.007	35.494	2.56 × 10^−9^
#16	28,509,237-28,525,062	Upstream	TTTGTCCCTACAGGCCTTT	0.500, 0.061	22.381	2.24 × 10^−6^
#23	28,629,162-28,640,811	Upstream	GAACTAATACCGA	0.250, 0.007	35.344	2.76 × 10^−9^

**Table 3:** Associations with “having 10 or more outpatient surgeries” considering the haplotypic phase of non-coding regulatory haplotype (linkage disequilibrium (LD) Block #16) and Val122Ile mutation in the carriers-only sample (*n* = 152).

Block #16 Haplotype	V122I (G > A)	Case-Control Frequencies	Chi Square	*p* Value
CTTGTTCATACAGGCTATT	A	0.286, 0.417	0.551	0.4581
CTTGTTCATACAGGCTATT	G	0.214, 0.300	0.275	0.5998
TAAACCACCGTACATCTGC	G	0.000, 0.075	0.646	0.4215
TAAACCACCGTGCATCTGC	G	0.000, 0.066	0.568	0.4509
TTTGTCCCTACAGGCCTTT	A	0.339, 0.048	12.482	4 × 10^−4^
TAAACCACCGTACATCTG	A	0.000, 0.033	0.276	0.5996
TTTGTCCCTACAGGCCTTT	G	0.161, 0.013	10.55	0.0012
CTTGTTCATACAGGCCTTT	G	0.000, 0.017	0.137	0.7117

## Supplementary Materials

The following are available online at https://www.mdpi.com/2077-0383/8/2/269/s1, **Figure S1:** Genomic region investigated in the haplotype association analysis (GRCh37/hg19 chr 18: 28,171,770–30,174,635). The regions highlighted in light blue indicate the non-coding haplotypes identified as significant. *TTR* gene region is highlighted in red. **Table S1:** Variants included in the haplotype association analysis. Information regarding the imputation quality (Info score) in Illumina HumanCoreExome array (HCE) and Illumina HumanOmni1-Quad v1.0 microarray (OMNI) is also reported. **Table S2:** Details of the association analysis between Val122Ile and traits related to medical history. Beta values are reported as effect size for linear regression models. Odds Ratios are reported as effect size for logistic regression models. **Table S3**: LD blocks identified in the genomic regions investigated in the haplotype association analysis (GRCh37/hg19 chr 18: 28,171,770–30,174,635). The rsId of the variants are reported in Table S1. **Table S4**: Association results of all haplotypes tested with respect to “having 10 or more outpatient surgeries” among Val122Ile carriers. **Table S5**: Functional annotation (Haploreg v4.1) of non-coding haplotypes identified as significantly associated with “having 10 or more outpatient surgeries” among Val122Ile carriers.
