# Supplementary material for: Increased Risk of Multiple Outpatient Surgeries in African-American Carriers of Transthyretin Val122Ile Mutation Is Modulated by Non-Coding Variants
Source: J Clin Med. 2019 Feb 22;8(2):269. doi: 10.3390/jcm8020269 (PMC6406512; doi:10.3390/jcm8020269)
Supplement: Supplementary file 1 [file jcm-08-00269-s001.pdf]

Journal of
Clinical Medicine

MDPI

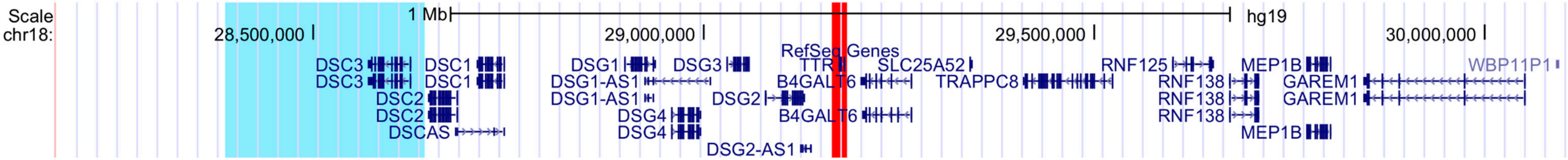

Figure S1. Genomic region investigated in the haplotype association analysis (GRCh37/hg19 chr 18: 28,171,770–30,174,635). The regions highlighted in light blue indicate the non-coding haplotypes identified as significant; the TTR gene region is highlighted in red.

Table S1. Variants included in the haplotype association analysis. Information regarding the imputation quality (Info score) in Illumina HumanCoreExome array (HCE) and Illumina HumanOmni1-Quad v1.0 microarray (OMNI) is also reported.

Order ID	rsId	Chr18 Location (bp)	Info (HCE)	Info (OMNI)
1	rs72927386	28171770	0.97	0.985
2	rs1038091	28171955	0.983	0.989
3	rs1038090	28171964	0.984	0.99
4	rs72927387	28171983	0.97	0.985
5	rs2542738	28172558	0.981	0.988
6	rs2617924	28172572	0.981	0.988
7	rs2542739	28172599	0.981	0.988
8	rs2729434	28172784	0.984	0.992
9	rs3110080	28172947	0.968	0.981
10	rs3110079	28172955	0.969	0.982
11	rs3095762	28173026	0.983	0.992
12	rs3101752	28173051	0.985	0.993
13	rs62079498	28173085	0.965	0.974
14	rs2927773	28173274	0.98	0.983
15	rs62079529	28173640	0.986	0.993
16	rs2927772	28174201	0.996	0.995
17	rs2927771	28174368	0.982	0.995
18	rs3101751	28174573	0.997	0.996
19	rs2617923	28175228	0.998	0.998
20	rs2542752	28175644	0.981	0.998
21	rs62079532	28175844	0.984	0.999

22	rs57982380	28176374	0.98	0.998
23	rs58478352	28176446	0.98	0.998
24	rs17797075	28176688	0.98	0.999
25	rs2678792	28177167	0.99	0.999
26	rs2678791	28177282	0.974	0.998
27	rs2617920	28178048	0.986	0.998
28	rs2052366	28178435	0.982	0.996
29	rs2909280	28180273	0.958	0.997
30	rs2945544	28180636	0.955	0.989
31	rs2617917	28180865	0.962	0.981
32	rs9948760	28184814	0.956	0.993
33	rs9959926	28184831	0.957	0.993
34	rs1382401	28186425	0.952	0.997
35	18:28188230	28188230	0.956	0.996
36	18:28188231	28188231	0.958	0.998
37	rs1478539	28188930	0.961	0.998
38	rs1478540	28189003	0.952	0.99
39	rs28580004	28189640	0.967	0.998
40	rs1478541	28190192	0.97	0.998
41	rs9972980	28190628	0.966	0.994
42	rs11875113	28191099	0.978	0.998
43	rs8083391	28191545	0.98	0.996
44	rs78253403	28191546	0.98	0.996
45	rs6508698	28192811	0.982	0.996
46	rs9947476	28193012	0.998	0.999

47	rs9963082	28193502	0.963	0.992
48	rs9951965	28194505	0.995	0.996
49	rs9966738	28195191	0.992	0.995
50	rs16948309	28200204	0.988	0.995
51	rs28620449	28201341	0.988	0.994
52	rs1115927	28208338	0.973	0.992
53	rs1115928	28208468	0.987	0.995
54	rs2542757	28209612	0.993	0.995
55	rs1351156	28211695	0.963	0.992
56	rs2729424	28214185	0.984	0.989
57	rs2617910	28215742	0.973	0.981
58	rs1788982	28251051	0.958	0.977
59	rs9304562	28252948	0.993	0.993
60	rs1943550	28263732	0.984	0.989
61	rs1893689	28265187	0.979	0.991
62	rs1943554	28271078	0.96	0.975
63	rs1943556	28276573	0.954	0.987
64	rs2733133	28277898	0.97	0.992
65	rs12607549	28281202	0.962	0.986
66	rs2678802	28282553	0.995	0.998
67	rs2678800	28282867	0.971	0.994
68	rs2733131	28283096	0.993	0.996
69	rs1943561	28284325	0.972	0.994
70	rs2678799	28284839	0.97	0.991
71	rs1943563	28285369	0.968	0.989

72	rs2850282	28286020	0.967	0.988
73	rs2678798	28286552	0.965	0.986
74	rs1943564	28287043	0.963	0.985
75	rs1943565	28287211	0.963	0.985
76	rs6508700	28287929	0.977	0.979
77	rs2678797	28288045	0.961	0.983
78	rs2850283	28288070	0.961	0.982
79	rs2733130	28288968	0.975	0.978
80	rs2733129	28289616	0.956	0.98
81	rs2678795	28290604	0.955	0.978
82	rs2678794	28290624	0.969	0.975
83	rs2733128	28291546	0.952	0.976
84	rs1943568	28291888	0.952	0.976
85	rs1943569	28292008	0.952	0.975
86	rs2850285	28292291	0.951	0.975
87	rs2678793	28292472	0.95	0.973
88	rs1943570	28292519	0.959	0.972
89	rs1943571	28292707	0.951	0.974
90	rs1943572	28292819	0.951	0.974
91	rs1943573	28292940	0.95	0.974
92	rs9966963	28299262	0.971	0.983
93	rs2187340	28299486	0.973	0.984
94	rs2187341	28299488	0.973	0.984
95	rs10163960	28299974	0.991	0.993
96	rs11664075	28303121	0.992	0.995

97	rs2850286	28304370	0.976	0.986
98	rs1943576	28305035	0.971	0.988
99	rs11083414	28308671	0.978	0.986
100	rs11083415	28308975	0.967	0.985
101	rs9948189	28309445	0.98	0.988
102	rs9950904	28309562	0.964	0.983
103	rs8088789	28309880	0.977	0.986
104	rs2591106	28309918	0.969	0.982
105	rs8090735	28309921	0.976	0.985
106	rs13381994	28311298	0.962	0.982
107	rs9955929	28311798	0.974	0.985
108	rs55681480	28312444	0.96	0.981
109	rs28799037	28312598	0.971	0.983
110	rs4616356	28312844	0.971	0.983
111	rs115351827	28313506	0.958	0.98
112	rs7237101	28313532	0.958	0.98
113	rs117093941	28313670	0.968	0.981
114	rs7240098	28314117	0.957	0.98
115	rs55739334	28314147	0.968	0.982
116	rs114649670	28314527	0.967	0.982
117	rs8084322	28315420	0.966	0.98
118	rs8085286	28315759	0.965	0.98
119	rs62086422	28316561	0.962	0.978
120	rs12457567	28316742	0.953	0.978
121	rs2733120	28318372	0.982	0.991

122	rs2733121	28319512	0.97	0.983
123	rs2591103	28320886	0.988	0.994
124	rs2097049	28321524	0.951	0.975
125	rs62086423	28322256	0.956	0.971
126	rs58351269	28322750	0.979	0.988
127	rs59639202	28322938	0.976	0.986
128	rs9635999	28350472	0.969	0.985
129	rs4598986	28350754	0.967	0.987
130	rs9947312	28351639	0.973	0.987
131	rs9962487	28351738	0.969	0.989
132	rs4799299	28352763	0.975	0.989
133	rs56027591	28353385	0.972	0.991
134	rs2733135	28354062	0.966	0.986
135	rs3898132	28354245	0.973	0.992
136	rs73952509	28355496	0.988	0.994
137	rs28510557	28355510	0.987	0.993
138	rs62086430	28360080	0.972	0.981
139	rs113512125	28360188	0.977	0.982
140	rs2591122	28361369	0.955	0.975
141	rs2733142	28364169	0.967	0.982
142	rs2733143	28364896	0.98	0.989
143	rs1943535	28366712	0.969	0.986
144	rs2591120	28367357	0.992	0.995
145	rs11660936	28367488	0.951	0.997
146	rs2591119	28367748	0.998	0.999

147	rs4121810	28368424	0.952	0.998
148	rs2733145	28368838	0.981	0.991
149	rs2733147	28369350	0.987	0.992
150	rs2733148	28369388	0.986	0.992
151	rs2733149	28369546	0.987	0.992
152	rs2733150	28370051	0.955	0.985
153	rs2733151	28370084	0.983	0.991
154	rs2591117	28370223	0.98	0.99
155	rs2591116	28370584	0.977	0.989
156	rs9955034	28371047	0.953	0.989
157	rs2105759	28372225	0.953	0.984
158	rs56271253	28373385	0.961	0.99
159	rs2733152	28373617	0.956	0.987
160	rs2591113	28377098	0.952	0.985
161	rs2850269	28381879	0.956	0.998
162	rs1943542	28384392	0.98	0.995
163	rs62088960	28385092	0.971	0.994
164	rs9951783	28385120	0.994	0.997
165	rs4799300	28385705	0.981	0.996
166	rs4799555	28385825	0.983	0.996
167	rs2591112	28386114	0.972	0.993
168	rs62088961	28387393	0.972	0.995
169	rs8098472	28387576	0.984	0.996
170	rs2850275	28387915	0.954	0.991
171	rs28644250	28388092	0.982	0.991

172	rs4627446	28388909	0.994	0.998
173	rs9950324	28388930	0.986	0.998
174	rs2000806	28388999	0.996	0.999
175	rs2000805	28389172	0.952	0.99
176	rs1943540	28390156	0.991	0.996
177	rs4799557	28390548	0.991	0.997
178	rs17711466	28390719	0.974	0.996
179	rs9962586	28391602	0.968	0.993
180	rs17711533	28391815	0.972	0.994
181	rs9304563	28392088	0.974	0.994
182	rs61048798	28392941	0.971	0.994
183	rs1369360	28393310	0.97	0.994
184	rs2000803	28394027	0.977	0.994
186	rs17711559	28394552	0.956	0.986
187	rs2572366	28396016	0.971	0.994
188	rs2591110	28397548	0.994	0.999
189	rs62088993	28401067	0.954	0.99
190	rs16948494	28443365	0.994	0.996
191	rs76367024	28444079	0.993	0.996
192	18:28445495	28445495	0.986	0.994
193	rs72930840	28445496	0.955	0.972
194	rs13381175	28447198	0.981	0.99
195	rs112558049	28448660	0.979	0.993
196	rs111680026	28448905	0.977	0.991
197	rs58278716	28449829	0.97	0.987

198	rs59095978	28450060	0.976	0.994
199	rs77038995	28450601	0.961	0.974
200	rs16948504	28451397	0.974	0.994
201	rs7240476	28453252	0.956	0.99
202	rs56352067	28456564	0.984	0.993
203	rs1532283	28459279	0.98	0.991
204	rs68134899	28463492	0.974	0.988
205	rs9953253	28470934	0.967	0.998
206	rs113442636	28471360	0.967	0.998
207	rs10164247	28475454	0.968	0.996
208	rs111561958	28478672	0.982	0.991
209	rs72918851	28478688	0.983	0.991
210	rs28546651	28480377	0.989	0.993
211	rs16960912	28480515	0.985	0.99
212	rs72918852	28481454	0.995	0.997
213	rs16948575	28481528	0.991	0.993
214	rs28522789	28481625	0.993	0.994
215	rs3855662	28482715	0.976	0.986
216	18:28483078	28483078	0.971	0.983
217	18:28483079	28483079	0.97	0.983
218	rs66663800	28484027	0.966	0.981
219	rs17798291	28484306	0.96	0.976
220	rs72918853	28484316	0.959	0.976
221	rs9957088	28509237	0.977	0.981
222	rs28758452	28510443	0.972	0.985

223	rs62089046	28510823	0.971	0.985
224	rs8092513	28511453	0.978	0.988
225	rs2082771	28513101	0.988	0.996
226	rs2420204	28513787	0.992	0.995
227	rs2117612	28514051	0.954	0.984
228	rs2117613	28514614	0.994	0.996
229	rs58982770	28517361	0.957	0.986
230	rs1602894	28517938	0.988	0.997
231	rs10163717	28518725	0.984	0.997
232	rs2082773	28519715	0.953	0.974
233	rs10163924	28520326	0.988	0.996
234	rs12326186	28522317	0.997	1
235	rs3910339	28523022	0.963	0.987
236	rs9962221	28523651	0.969	0.989
237	rs9952824	28524281	0.966	0.988
238	rs7242206	28524832	0.984	0.991
239	rs1865515	28525062	0.982	0.989
241	rs4799558	28544172	0.965	0.994
242	rs1313579	28581442	0.951	0.993
243	rs1314310	28582451	0.975	0.994
244	rs1313581	28583870	0.962	0.99
245	rs1908138	28583938	0.99	0.994
246	rs1313582	28583981	0.972	0.992
247	rs7227115	28585638	0.991	0.998
248	rs66811121	28585784	0.989	0.997

249	rs1313585	28586423	0.968	0.995
251	rs8087664	28586546	0.975	0.995
252	rs1789068	28586620	0.966	0.995
253	rs2047083	28587180	0.966	0.995
254	rs7229311	28589490	0.964	0.993
255	rs276911	28596566	0.979	0.994
256	rs276914	28599666	0.984	1
257	rs276915	28599981	0.995	0.999
258	rs276916	28600161	0.994	0.999
259	rs276917	28600246	0.984	0.999
261	rs9807290	28601044	0.95	0.996
262	rs276920	28601160	0.985	0.998
263	rs8091492	28601208	0.951	0.986
264	rs1790700	28601730	0.985	0.998
265	rs1617054	28601735	0.984	0.993
266	rs1313596	28601960	0.985	0.998
269	rs276923	28602945	0.986	0.999
278	rs1595357	28604104	0.968	0.996
283	rs166724	28605492	0.987	0.996
284	rs202439	28605715	0.992	0.996
287	rs1313594	28606110	0.992	0.995
291	rs276945	28606768	0.995	0.996
292	rs169347	28606778	0.995	0.996
293	rs1313593	28606825	0.995	0.996
301	rs115237731	28609072	0.957	0.999

303	rs276941	28609220	0.999	0.999
306	rs276940	28609770	0.992	0.996
315	rs74849509	28610856	0.961	0.994
316	rs2850324	28610877	0.982	0.991
317	rs276939	28610887	0.996	0.996
318	rs276937	28611061	0.999	0.999
320	rs114393445	28611460	0.975	0.987
321	rs116543292	28611665	0.974	0.986
322	rs115300587	28611737	0.973	0.986
323	rs115465507	28611744	0.973	0.986
324	rs147235980	28611961	0.973	0.985
326	rs151272810	28612344	0.971	0.984
327	rs116639442	28612351	0.964	0.976
328	rs114203680	28612777	0.968	0.994
329	rs116467185	28612832	0.963	0.994
330	rs115787862	28612891	0.97	0.983
331	rs114097400	28613064	0.97	0.983
332	rs115114836	28613198	0.97	0.983
333	rs76313124	28613206	0.969	0.983
334	rs175777	28613821	0.981	0.986
335	rs1313599	28613837	0.983	0.987
336	rs166725	28613983	0.983	0.987
340	rs115137609	28614719	0.967	0.981
342	rs142847351	28614827	0.967	0.981
351	rs151081226	28615993	0.98	0.995

359	rs276935	28616855	0.984	0.991
360	rs28657717	28616926	0.992	0.995
362	rs276934	28617047	0.959	0.988
363	rs12955873	28617053	0.987	0.989
366	rs116465993	28617402	0.987	0.998
368	rs1789046	28617483	0.984	0.991
369	rs1790666	28617552	0.989	0.993
370	rs150816573	28617842	0.967	0.994
373	rs78981636	28618811	0.993	0.999
374	rs276932	28618926	0.994	0.998
375	rs276931	28619690	0.991	0.997
377	rs1816635	28619806	0.996	0.998
378	rs276930	28620042	0.994	0.996
379	rs79395750	28620326	0.996	0.998
380	rs276929	28620394	0.992	0.995
381	rs114101336	28620630	0.99	0.998
382	rs276928	28620632	0.993	0.996
384	rs2245035	28620676	0.993	0.996
385	rs1790665	28620679	0.993	0.996
386	rs276927	28620721	0.993	0.996
388	rs1586961	28621211	0.998	1
389	rs1586960	28621238	0.999	1
390	rs1586958	28621423	1	1
392	rs41465650	28621619	0.999	0.999
395	rs276925	28622048	0.992	0.996

396	rs276924	28622524	0.982	0.987
398	rs28547150	28622971	0.996	0.999
399	rs28688431	28623001	0.999	0.999
402	rs3810016	28623782	0.994	0.996
403	rs3810015	28623794	0.993	0.996
405	rs3810011	28624553	0.994	1
406	rs10502556	28624817	0.966	0.993
409	rs11875739	28626312	0.98	0.997
410	rs1595349	28626380	0.965	0.99
412	rs116454306	28629162	0.977	0.982
414	18:28633522	28633522	0.954	0.979
415	rs1658100	28635377	0.967	0.984
416	rs11081678	28635776	0.969	0.995
417	rs1790696	28635877	0.966	0.988
418	rs1658101	28636616	0.971	0.988
419	rs74564112	28636928	0.95	0.972
420	rs1595355	28637196	0.974	0.989
421	rs1790695	28638832	0.992	0.995
422	rs114554358	28639330	0.975	0.982
423	rs116561824	28639449	0.952	0.986
424	rs1595356	28639929	0.993	0.998
426	rs16961188	28640811	0.977	0.987
427	rs1658102	28641443	0.99	0.994
428	rs1790694	28642129	0.965	0.989
429	rs2019624	28642502	0.97	0.99

430	rs4559974	28643071	0.967	0.986
431	rs75391368	28644656	0.958	0.98
433	rs79887529	28648423	0.955	0.962
434	rs75543658	28648551	0.955	0.962
436	rs1893961	28649215	0.993	0.997
437	rs115511898	28649488	0.951	0.96
438	rs4799304	28652480	0.962	0.983
439	rs67808411	28655873	0.993	0.994
441	rs1790702	28662776	0.955	0.978
442	18:28663303	28663303	0.954	0.978
443	rs74513256	28663505	0.959	0.982
444	rs62722063	28680371	0.957	0.968
445	rs1437604	28686766	0.987	0.992
446	rs12960042	28692016	0.976	0.987
447	rs1595358	28692824	0.984	0.99
448	rs73954505	28693421	0.96	0.98
449	rs73954506	28693423	0.96	0.98
450	rs1790669	28693553	0.964	0.989
451	rs1790670	28693601	0.964	0.989
452	rs1626700	28693728	0.964	0.989
453	rs1790671	28693779	0.966	0.99
454	rs1790673	28694052	0.964	0.989
455	rs1790674	28694215	0.964	0.989
456	rs1790675	28694379	0.962	0.987
457	rs1658130	28694498	0.966	0.99

458	rs1790676	28694951	0.966	0.991
459	rs1789042	28694997	0.963	0.99
460	rs1658131	28695061	0.963	0.99
461	rs73415549	28695950	0.973	0.992
462	rs1790677	28696013	0.983	0.992
463	rs1790678	28696381	0.983	0.992
464	rs58702329	28696478	0.967	0.995
465	rs12455322	28696569	0.984	0.993
466	rs73415554	28696718	0.968	0.995
467	rs55982246	28697369	0.957	0.998
468	rs1790688	28697594	0.984	0.994
469	rs1658134	28697719	0.967	0.995
470	rs16961254	28697994	0.959	0.999
471	rs114746645	28698608	0.953	0.988
472	rs1626413	28699514	0.984	0.993
473	rs16961256	28699538	0.992	1
474	rs73954515	28700409	0.995	1
475	rs73415572	28700649	0.983	0.998
476	rs73415573	28700900	0.981	1
477	rs4799563	28701645	0.969	0.996
478	rs4799564	28701708	0.97	0.996
479	rs59683603	28701816	0.965	0.998
480	rs16961260	28702091	0.972	0.996
481	rs11874340	28702375	0.989	0.996
482	rs79229352	28702744	0.977	0.995

483	rs1658135	28702856	0.97	0.996
484	rs58911015	28703152	0.962	0.998
485	rs58689925	28703236	0.974	0.999
486	rs59270371	28705533	0.968	0.999
487	rs1790691	28705604	0.952	0.991
488	rs73415585	28706234	0.971	0.999
489	rs73421614	28706519	0.969	0.999
490	rs989307	28706947	0.968	0.996
491	rs8086137	28707415	0.968	0.999
492	rs2156761	28708361	0.959	1
493	rs8095354	28709604	0.961	0.999
494	rs76575208	28709957	0.96	0.996
495	rs77457995	28711056	0.958	0.994
496	rs16961322	28711206	0.958	0.994
497	rs16961328	28711303	0.953	0.997
498	rs16961331	28711445	0.952	0.997
499	rs16961333	28711504	0.953	0.994
500	rs1658112	28713111	0.986	0.999
502	rs1658111	28715716	0.962	0.995
503	rs12455973	28721882	0.994	0.994
504	rs12458870	28721913	0.995	0.996
505	rs12458919	28721922	0.996	0.997
506	rs34064740	28722644	0.996	0.998
507	18:28723344	28723344	0.989	0.992
508	18:28723349	28723349	0.989	0.992

509	18:28723354	28723354	0.989	0.992
510	rs12454687	28723469	0.993	0.996
511	rs2577072	28723596	0.993	0.996
512	rs12454773	28723832	0.98	0.99
513	rs12454734	28723835	0.992	0.994
514	rs7237666	28724105	0.991	0.994
515	rs7236502	28724161	0.991	0.994
516	rs7236955	28724268	0.974	0.987
517	rs7236673	28724320	0.984	0.992
518	rs7238480	28724665	0.982	0.99
519	rs7238483	28724671	0.982	0.99
520	rs7237676	28724814	0.976	0.988
521	rs2851994	28724934	0.967	0.982
522	rs12606538	28726317	0.97	0.978
523	rs12606478	28726348	0.97	0.978
524	rs12604470	28726350	0.97	0.978
525	rs12606557	28726422	0.97	0.978
526	rs2577070	28726499	0.961	0.977
527	rs61706330	28726618	0.961	0.977
528	rs72926566	28726884	0.961	0.978
529	rs72926569	28727043	0.961	0.978
530	rs75939428	28727376	0.958	0.979
531	rs77383140	28727466	0.958	0.979
532	rs1982162	28727833	0.958	0.98
533	rs1982161	28727959	0.959	0.981

534	rs11874325	28728198	0.959	0.981
535	rs11877211	28728268	0.959	0.982
536	rs11877142	28728281	0.959	0.982
537	rs3213883	28728411	0.959	0.982
538	rs12608327	28729104	0.955	0.979
539	rs12608367	28729168	0.959	0.983
540	rs12606277	28729197	0.966	0.983
541	rs59703079	28729204	0.967	0.984
542	rs58434527	28729323	0.969	0.986
543	rs59043085	28729458	0.969	0.986
544	rs17800231	28729576	0.969	0.986
545	rs17727175	28729754	0.97	0.987
546	rs79681568	28729933	0.969	0.988
547	rs77220749	28729943	0.97	0.988
548	rs77360088	28729947	0.97	0.988
549	18:28730112	28730112	0.954	0.974
550	rs77300285	28730599	0.971	0.991
551	rs72926587	28730634	0.972	0.991
552	rs3786169	28730816	0.972	0.992
553	rs12606830	28731916	0.966	0.994
554	chr18:28732135:I	28732135	0.967	0.999
555	rs12606879	28732281	0.982	0.997
556	rs12606944	28732330	0.975	0.999
557	rs17727199	28732443	0.966	1
558	18:28732877	28732877	0.965	0.999

559	rs11874090	28733202	0.964	0.998
560	rs9952225	28733437	0.987	0.994
561	rs7238415	28733798	0.973	0.993
563	rs2577066	28735118	0.978	0.999
564	rs2741004	28735423	0.977	0.995
565	rs2741003	28735454	0.979	0.997
566	rs2852000	28735456	0.984	0.998
567	rs2577064	28735534	0.987	0.999
568	rs2851999	28735679	0.978	1
569	rs56143587	28735766	0.987	1
570	rs2164766	28736457	0.975	0.994
572	rs2118343	28736869	0.979	0.997
573	rs2246736	28737198	0.986	1
574	rs2118342	28737299	0.984	0.997
575	rs28630791	28737320	0.97	0.998
576	rs6506884	28737756	0.988	0.998
577	rs6506885	28737770	0.988	0.998
578	rs2741000	28737883	0.982	0.997
579	rs6506886	28737916	0.976	0.994
580	rs6506887	28738014	0.99	0.999
581	rs6506888	28738049	0.986	0.997
582	rs6506889	28738059	0.991	0.999
583	rs7245261	28738104	0.989	0.998
584	rs6506890	28738118	0.991	0.999
585	rs7245164	28738187	0.991	0.999

586	rs9956307	28738332	0.992	0.998
587	rs3786168	28738397	0.993	0.999
588	rs113505305	28739048	0.977	0.985
589	18:28739105	28739105	0.979	0.982
590	18:28739108	28739108	0.979	0.982
591	18:28739109	28739109	0.996	0.998
593	rs61248090	28739771	0.973	0.996
594	rs8093017	28740608	0.973	0.997
595	rs1370450	28741821	0.982	0.992
597	rs6506891	28741908	0.985	0.997
598	rs2217989	28742058	0.981	0.995
599	rs1816633	28742231	0.984	0.992
600	rs1816632	28742268	0.987	0.995
601	rs73406930	28742601	0.971	0.996
603	rs9955430	28743678	0.987	0.994
604	rs9944812	28743705	0.988	0.994
606	rs4325646	28744391	0.984	0.994
607	rs8090853	28744958	0.974	0.995
608	rs8091932	28745174	0.951	0.993
609	rs8096368	28746634	0.985	0.994
610	rs9960487	28748147	0.984	0.991
611	rs9948366	28748289	0.998	0.998
612	rs1437612	28748466	0.998	0.999
613	rs1437611	28748591	0.998	0.997
614	rs1437610	28748625	0.999	0.999

615	rs1437609	28748818	0.985	0.995
617	rs976750	28749085	0.999	0.999
618	rs976749	28749481	0.997	0.998
619	rs4344831	28749716	0.995	0.997
620	rs4359521	28749783	0.992	0.995
621	rs4361001	28749839	0.994	0.997
623	rs9955032	28750483	0.991	0.996
625	rs2118341	28751059	0.989	0.995
626	rs2164765	28751189	0.97	0.995
627	rs6506892	28751774	0.968	0.994
628	rs9947328	28753215	0.98	0.993
629	rs1437608	28754974	0.984	0.992
630	rs1437607	28758389	0.989	0.994
631	rs1866106	28759539	0.991	0.995
632	rs1866105	28759614	0.986	0.994
633	rs11662538	28761676	0.993	0.997
634	rs4799566	28762480	0.981	0.99
635	rs991482	28764624	0.994	0.997
636	rs951298	28765917	0.974	0.987
637	rs2919996	28769637	0.96	0.986
638	rs8098864	28803575	0.951	0.978
639	rs9960813	28808684	0.952	0.977
641	rs7226461	28817662	0.958	0.987
642	rs8088637	28818616	0.968	0.989
643	rs8087786	28818851	0.971	0.99

644	rs8088973	28818858	0.984	0.991
645	rs8091715	28818913	0.973	0.991
646	rs8091871	28819199	0.99	0.994
647	rs35229932	28819949	0.983	0.996
648	rs11659665	28823180	0.972	0.985
649	rs6506903	28825084	0.972	0.995
650	rs4408611	28825176	0.973	0.995
651	rs8089598	28827706	0.988	0.997
652	rs7231460	28832187	0.981	0.999
653	rs62087700	28834165	0.985	0.998
654	rs4517864	28835308	0.983	0.996
655	rs2162274	28837058	0.99	0.998
657	rs34530511	28838039	0.99	0.999
658	rs4539673	28838769	0.997	0.999
659	rs8087103	28839294	0.985	0.995
660	rs12606722	28839528	0.996	0.998
662	rs6506904	28840202	0.995	0.996
663	rs7235384	28840259	0.995	0.996
664	rs62087701	28840543	0.994	0.995
665	rs62087702	28840557	0.982	0.992
666	rs62087703	28840645	0.993	0.995
667	rs12959058	28841259	0.99	0.993
668	rs4598984	28841397	0.987	0.992
669	rs17658464	28842181	0.954	0.981
670	rs35136508	28842206	0.958	0.983

672	rs2912318	28844359	0.953	0.975
673	rs62087706	28848162	0.955	0.992
674	rs76634529	28848478	0.956	0.993
675	rs9951341	28849733	0.958	0.983
676	rs2919981	28850887	0.979	0.984
677	rs35107698	28851076	0.953	0.984
678	rs16961601	28852162	0.956	0.988
679	rs62087754	28852522	0.962	0.988
680	rs16961605	28852591	0.959	0.991
681	rs16961609	28853542	0.96	0.991
682	rs7234474	28854117	0.961	0.991
684	rs11660638	28854472	0.957	0.982
686	rs1365290	28854740	0.977	0.991
689	rs62087755	28859975	0.99	0.992
690	rs62087756	28860547	0.987	0.991
691	rs74796545	28860728	0.986	0.991
692	rs56697971	28860866	0.985	0.988
694	rs59592286	28861384	0.986	0.99
698	rs55778425	28862014	0.976	0.983
699	rs56088904	28862024	0.971	0.982
700	rs35287825	28862612	0.964	0.988
701	rs11663919	28862956	0.962	0.983
702	rs11663924	28862965	0.976	0.983
704	rs116822607	28863621	0.978	0.985
705	rs114848551	28863659	0.962	0.984

706	rs61691923	28863672	0.978	0.985
707	rs55965644	28863902	0.978	0.984
708	rs111513798	28864173	0.969	0.983
709	rs149280789	28864461	0.967	0.983
710	rs7241874	28864700	0.978	0.985
711	rs67550130	28864969	0.964	0.988
712	rs7227412	28865007	0.976	0.984
713	rs28786993	28865203	0.954	0.976
714	rs35223713	28865430	0.978	0.985
715	rs2912341	28865525	0.98	0.986
716	rs142217576	28865796	0.973	0.985
717	rs11660200	28866077	0.982	0.988
718	rs11873223	28866470	0.97	0.99
720	rs11081685	28866632	0.983	0.989
721	rs11873281	28866652	0.978	0.987
723	rs62088670	28867238	0.97	0.977
724	18:28867489	28867489	0.97	0.994
726	rs62088672	28868262	0.97	0.978
727	rs9967347	28868312	0.972	0.987
728	rs9955289	28868400	0.987	0.993
729	rs7245063	28868486	0.988	0.993
730	rs9955239	28868514	0.988	0.993
732	rs7229763	28868901	0.988	0.993
733	rs116508586	28868910	0.961	0.986
734	rs7229799	28868977	0.989	0.994

735	rs7230092	28869167	0.99	0.995
737	rs2919984	28869365	0.989	0.995
738	rs6506908	28869598	0.991	0.996
739	rs12957591	28869730	0.992	0.996
740	rs2912339	28869782	0.986	0.991
741	rs12958464	28869788	0.987	0.998
742	rs2912338	28869954	0.989	0.995
743	rs9961275	28870134	0.993	0.997
744	rs9961633	28870205	0.993	0.997
746	rs62088673	28871639	0.974	0.984
747	rs7231775	28871685	0.996	0.998
748	rs2919985	28872033	0.999	0.999
749	rs58106182	28872053	0.983	0.992
750	rs62088674	28872137	0.969	0.981
751	rs62088675	28872387	0.998	0.999
752	rs11661048	28872653	0.996	0.999
753	rs11661104	28872852	0.999	1
754	rs9966267	28873255	0.999	0.999
755	rs2912335	28873910	0.993	0.996
757	rs62088676	28875020	0.972	0.986
758	rs73413256	28875112	0.998	1
759	rs7234921	28875268	0.998	1
760	rs6506910	28875628	0.993	0.996
761	rs73413259	28876054	0.996	0.998
763	rs9967293	28877283	0.991	0.995

764	rs56748528	28877752	0.992	0.994
765	rs9944661	28877753	0.99	0.994
766	rs9947250	28878231	0.992	0.995
767	rs6506912	28878488	0.988	0.994
768	rs9947771	28878783	0.986	0.992
769	rs1895902	28878874	0.989	0.994
771	rs8092044	28879612	0.981	0.996
772	rs28566128	28879750	0.989	0.992
773	rs9953981	28880539	0.99	0.996
774	rs9953985	28880565	0.995	0.997
775	rs11876962	28881530	0.993	0.998
776	rs59593146	28881763	0.98	0.997
777	rs7244719	28882630	0.993	0.999
780	18:28883398	28883398	0.968	0.983
781	18:28883402	28883402	0.975	0.993
782	rs2912334	28883507	0.99	0.998
783	rs74342376	28883883	0.977	0.994
784	rs62088713	28884179	0.977	0.995
785	rs2919989	28884849	0.98	0.997
786	rs9304080	28885580	0.997	1
788	rs9304081	28886603	0.995	1
789	rs2919990	28886708	0.989	1
791	rs9973105	28886950	0.984	0.999
793	rs11081686	28887350	0.991	1
795	rs62088714	28888132	0.971	0.999

796	rs9304082	28888191	0.991	1
798	rs62088715	28888501	0.963	0.99
801	rs9973141	28888838	0.987	0.999
802	rs62088716	28889126	0.962	0.99
804	rs62088717	28889255	0.968	0.999
806	rs9675787	28889503	0.987	1
807	rs9676194	28889517	0.987	1
808	rs9304083	28889601	0.985	0.999
809	rs9304084	28889616	0.984	0.999
810	rs9304085	28889617	0.984	0.999
811	rs76891338	28889647	0.965	0.999
812	rs9304086	28889666	0.985	0.999
813	rs9304087	28889709	0.985	1
814	rs62088718	28889760	0.966	0.999
815	rs9304088	28889794	0.986	1
816	rs9304089	28889918	0.985	1
817	rs2919991	28889957	0.966	0.999
818	rs8084591	28889997	0.985	1
819	rs58860406	28890224	0.969	0.998
820	rs6506913	28890319	0.984	1
821	rs8086438	28890520	0.964	1
822	rs8085334	28890581	0.964	1
823	rs79054887	28890633	0.964	1
825	rs76971536	28890702	0.963	1
826	rs2042719	28890742	0.963	1

827	rs2042718	28890891	0.963	1
828	rs62088719	28891146	0.963	1
829	rs62088720	28891260	0.963	1
830	rs9955212	28891379	0.982	0.998
831	rs77551135	28891380	0.962	1
833	18:28891450	28891450	0.951	0.995
834	18:28891451	28891451	0.962	1
835	18:28891452	28891452	0.962	1
836	18:28891470	28891470	0.955	0.997
837	18:28891471	28891471	0.955	0.997
838	rs17728319	28891492	0.962	1
839	rs35464390	28891634	0.951	0.993
840	rs17801363	28891746	0.961	1
841	rs17728344	28891786	0.961	1
842	rs35825997	28891814	0.961	1
843	rs34242053	28891822	0.961	1
844	rs34155384	28891877	0.958	0.997
845	rs34399320	28891905	0.961	1
846	rs35785362	28891928	0.961	1
847	rs34521598	28892049	0.958	0.995
848	rs35112075	28892056	0.958	0.995
849	rs34494197	28892090	0.956	0.994
850	rs35777719	28892111	0.956	0.994
851	rs35645103	28892121	0.956	0.994
852	rs35659634	28892132	0.956	0.994

853	rs2912333	28892149	0.972	0.997
854	rs9947616	28892300	0.978	0.995
855	rs17728376	28892392	0.963	1
856	rs62088722	28892669	0.962	0.999
857	rs62088723	28892678	0.962	0.999
858	rs62088724	28892859	0.962	0.999
859	rs17801417	28893027	0.961	0.998
860	rs62088725	28893098	0.961	0.998
861	rs17728400	28893167	0.961	0.998
862	rs74554925	28893274	0.951	0.989
863	rs77256035	28893299	0.95	0.988
864	rs78014241	28893331	0.953	0.99
865	rs76298804	28893346	0.953	0.989
866	rs115569576	28893379	0.952	0.988
867	rs79007086	28893409	0.954	0.991
868	rs2919992	28893511	0.967	0.987
869	rs2919993	28893546	0.961	0.982
870	rs2912331	28893552	0.961	0.982
871	rs2919994	28893556	0.961	0.982
872	rs7236078	28893617	0.971	0.994
873	rs111806992	28894319	0.974	0.988
874	rs4399610	28894634	0.99	0.995
875	rs77739592	28895527	0.984	0.995
876	rs9948210	28895978	0.988	0.996
877	rs16961639	28896040	0.961	0.995

878	rs11081687	28896207	0.964	0.996
879	rs7233743	28896713	0.989	0.997
880	rs3810008	28896759	0.99	0.997
881	rs3810007	28896842	0.997	0.998
882	rs6506914	28897536	0.991	0.999
883	rs1834582	28898201	1	1
885	rs41326652	28899031	0.99	0.995
886	rs7234662	28900001	0.974	0.995
887	rs7234854	28900048	0.972	0.995
888	rs7236309	28900264	0.971	0.995
889	rs62090169	28900285	0.976	0.997
890	rs7235354	28900328	0.97	0.994
891	rs56674356	28900521	0.993	0.992
892	rs12962355	28900577	0.97	0.995
893	rs12963302	28900634	0.969	0.994
894	rs12607370	28900664	0.969	0.994
895	rs77111379	28901014	0.993	0.994
896	rs9959406	28901032	0.979	0.995
897	rs9947296	28901124	0.98	0.994
898	rs9947444	28901153	0.98	0.994
900	rs6506915	28901476	0.964	0.994
901	rs8087621	28901520	0.978	0.994
902	rs8088418	28901524	0.975	0.994
903	rs8087410	28901574	0.976	0.994
904	rs6506916	28901792	0.96	0.994

905	rs8091579	28902080	0.972	0.994
906	rs8091934	28902197	0.97	0.994
907	rs8091847	28902270	0.969	0.993
908	rs4356544	28903015	0.959	0.995
909	rs10438909	28903525	0.961	0.995
910	rs10438930	28903620	0.952	0.995
911	rs35382145	28904311	0.951	0.994
912	rs77566965	28904514	0.992	0.998
913	rs11875510	28904634	0.952	0.996
914	rs11875560	28904643	0.952	0.996
915	rs74373716	28905826	0.988	0.999
916	rs11665531	28907489	0.981	0.998
917	rs16961644	28908542	0.984	0.999
918	rs16961648	28910868	0.96	0.996
919	rs16961651	28911606	0.978	0.999
920	rs59844732	28913023	0.964	0.998
922	rs16961653	28914593	0.976	0.994
923	rs77442324	28914731	0.966	0.998
924	rs77447539	28915134	0.976	0.998
925	rs11664391	28916121	0.976	0.998
926	rs9963076	28916182	0.993	0.998
927	rs16961657	28916649	0.985	0.998
928	rs11877765	28916920	0.994	0.998
929	rs16961673	28917050	0.995	0.998
930	18:28917223	28917223	0.991	0.996

931	18:28917224	28917224	0.991	0.996
932	rs9966811	28917693	0.987	0.999
933	rs9966741	28917781	0.996	0.999
934	rs9946148	28918085	0.997	0.999
935	rs9946771	28918628	0.997	0.998
936	rs9946887	28918818	0.994	0.991
937	rs114033658	28919434	0.973	1
938	rs72927192	28920081	0.971	0.987
940	rs144033617	28928364	0.953	0.999
941	18:28928530	28928530	0.964	0.984
942	18:28928758	28928758	0.958	0.979
943	18:28932552	28932552	0.971	0.998
944	rs59911099	28933901	0.972	0.996
945	rs59243474	28933949	0.974	0.996
947	rs59045214	28934074	0.981	1
948	rs57121909	28934083	0.982	1
949	rs60226049	28934087	0.98	0.996
950	rs61730308	28934298	0.993	0.993
951	rs16961692	28934621	0.99	1
952	rs1042754	28934973	0.994	1
954	rs16961701	28936397	0.997	0.995
956	rs2420351	28936765	0.995	0.997
957	rs2420352	28936799	0.994	0.997
959	rs114896788	28937676	0.992	0.989
960	rs115718557	28937685	0.992	0.987

965	rs2035039	28938611	0.962	0.99
967	rs11662033	28938836	0.956	0.987
970	rs35779708	28940916	0.974	0.987
972	rs12961685	28942168	0.974	0.996
973	rs4799568	28942743	0.981	0.987
974	rs80120566	28943042	0.986	0.997
976	rs111915032	28943425	0.986	0.998
978	rs111963400	28943560	0.986	0.999
979	rs111548626	28943903	0.985	0.999
980	rs113687559	28944048	0.97	0.99
981	rs150326762	28944726	0.974	0.99
982	rs138003434	28944763	0.974	0.99
983	rs143521766	28944785	0.976	0.997
984	rs12954896	28944891	0.991	0.998
985	rs7231515	28944902	0.972	0.989
986	rs7232986	28945001	0.979	0.998
987	rs7231890	28945195	0.961	0.985
988	rs7232040	28945254	0.971	0.989
989	rs7232342	28945315	0.97	0.989
990	rs7232205	28945347	0.97	0.989
991	rs12606955	28945605	0.993	0.995
992	rs113282628	28945732	0.969	0.988
993	rs74621378	28945757	0.968	0.988
994	rs112171508	28946055	0.967	0.988
995	rs1991290	28946211	0.951	0.999

996	rs12968911	28947261	0.989	0.996
997	rs7229363	28948249	0.967	0.995
998	rs7229528	28948340	0.967	0.995
999	18:28948458	28948458	0.966	0.993
1000	rs7231403	28948887	0.965	0.994
1001	rs111821122	28949096	0.951	0.988
1002	rs112614317	28949122	0.964	0.994
1003	rs115517732	28949453	0.962	0.994
1004	rs1941188	28949468	0.953	0.992
1005	rs112347158	28949494	0.961	0.994
1006	rs9950246	28949852	0.99	0.998
1007	rs12966245	28950283	0.979	0.997
1008	rs11081693	28951038	0.986	0.998
1009	rs12605371	28951475	0.986	0.998
1010	rs1426312	28952356	0.98	0.998
1011	rs1834583	28952429	0.984	0.998
1012	rs9945110	28953071	0.982	0.998
1013	18:28954816	28954816	0.978	0.994
1014	rs1031731	28955051	0.968	0.999
1015	rs1031732	28955160	0.968	0.999
1016	rs12458294	28955504	0.967	0.997
1017	rs1350729	28955782	0.966	0.996
1018	rs8095518	28955851	0.978	0.996
1019	rs8099619	28956322	0.972	0.995
1020	rs11875880	28957811	0.976	0.995

1023	rs4799569	28958981	0.979	0.995
1024	rs11081695	28959867	0.971	0.992
1025	rs1350730	28960622	0.974	0.993
1026	rs8084577	28961937	0.98	0.995
1027	rs9635916	28962230	0.967	0.992
1028	rs12607068	28963050	0.978	0.993
1030	rs9304093	28963946	0.986	0.995
1031	rs3859361	28964634	0.968	0.992
1032	rs3899889	28964679	0.981	0.994
1033	rs1813419	28964860	0.981	0.994
1034	rs8086756	28965921	0.992	0.997
1035	rs1460596	28966284	0.994	0.998
1036	rs1460597	28966518	0.995	0.998
1038	rs8092320	28967410	0.996	0.998
1040	rs7233718	28970147	0.963	0.994
1041	rs77163042	28976817	0.959	0.993
1042	rs111361453	28977934	0.963	0.994
1043	rs79028557	28981514	0.975	0.993
1044	rs74364504	28985022	0.974	0.972
1045	rs12966815	28989216	0.981	0.984
1046	rs7234288	28993501	0.97	0.969
1047	rs1460605	28994752	0.979	0.981
1048	rs4799571	28996627	0.968	0.974
1049	rs7351033	29022370	0.997	0.995
1050	rs6506923	29022973	0.993	0.995

1051	rs6506924	29023007	0.993	0.995
1052	rs10048228	29023539	0.978	0.993
1053	rs10048329	29023625	0.989	0.996
1054	rs10048348	29023943	0.993	0.995
1055	rs12185410	29024156	0.994	0.996
1056	rs12185303	29024329	0.996	0.997
1057	rs12185412	29024352	0.993	0.996
1058	rs958961	29024467	0.997	0.997
1059	rs724481	29024566	0.997	0.998
1060	rs7237765	29025068	0.997	0.999
1061	rs7236759	29025230	0.997	0.999
1062	rs7236769	29025257	0.993	0.997
1063	rs16961915	29025531	0.959	0.995
1064	rs10048282	29025644	0.995	0.999
1065	rs10048305	29025949	0.994	0.998
1066	rs28698782	29026346	0.985	0.991
1067	rs28438375	29026371	0.982	0.989
1068	rs28550181	29026373	0.982	0.989
1069	rs60448934	29026498	0.973	0.994
1070	rs16961919	29026915	0.988	0.995
1071	rs3907152	29027233	0.968	0.988
1072	rs10084010	29027745	0.952	0.972
1073	rs8089502	29028184	0.993	0.999
1074	rs1460601	29028877	0.988	0.997
1075	rs9964117	29029429	0.985	0.996

1076	rs116175575	29029430	0.984	0.996
1077	rs9953370	29029435	0.984	0.996
1078	rs9964450	29029589	0.969	0.985
1079	rs7240484	29029768	0.971	0.988
1080	rs8099530	29030210	0.977	0.989
1081	rs9956575	29030497	0.977	0.988
1082	rs9956859	29030796	0.975	0.986
1083	rs61567349	29031047	0.976	0.987
1084	rs16961938	29031073	0.976	0.986
1085	rs56069436	29031612	0.983	0.988
1086	rs57787597	29031737	0.95	0.972
1087	rs16961940	29031909	0.987	0.991
1088	rs11081697	29032417	0.992	0.995
1089	rs11081698	29032657	0.991	0.994
1090	rs11081699	29032670	0.991	0.995
1091	rs6506925	29033140	0.994	0.998
1092	rs78146305	29033874	0.964	0.976
1093	rs8092800	29033928	0.994	0.998
1094	rs8092559	29034003	0.978	0.981
1095	rs8093256	29034262	0.99	0.992
1096	rs8094212	29034304	0.963	0.983
1097	rs8093149	29034326	0.975	0.988
1098	rs113886503	29034756	0.993	0.995
1099	rs67790517	29034768	0.983	0.99
1100	rs112243414	29034775	0.992	0.995

1101	rs67611221	29035177	0.993	0.994
1102	rs60094806	29035438	0.98	0.989
1103	rs61344982	29035439	0.979	0.989
1104	rs75744095	29035506	0.966	0.974
1105	rs16961954	29035579	0.989	0.989
1106	rs116142350	29036166	0.966	0.973
1107	rs3848485	29036316	0.98	0.989
1108	rs68094756	29037416	0.965	0.991
1109	rs151291304	29037784	0.968	0.973
1110	rs1942009	29037814	0.958	0.984
1111	rs111286338	29037839	0.983	0.993
1112	rs112754332	29037872	0.985	0.994
1113	rs67423158	29037913	0.985	0.994
1114	rs72925127	29037921	0.985	0.994
1115	rs72925129	29038060	0.983	0.995
1116	rs7240573	29038263	0.99	0.998
1117	rs113791745	29056785	0.977	0.995
1118	rs7241107	29068666	0.963	0.993
1119	rs9941421	29069119	0.971	0.995
1120	rs1824477	29069799	0.978	0.996
1121	rs9304097	29070441	0.995	0.999
1122	rs6506928	29070824	0.997	1
1123	18:29071365	29071365	0.994	0.998
1124	rs1350731	29071505	0.995	0.999
1125	rs2219584	29071787	0.994	0.999

1126	rs9951843	29072968	0.963	0.969
1127	rs7238729	29073191	0.963	0.969
1128	rs4799573	29075273	0.97	0.975
1129	rs7232282	29075793	0.972	0.977
1130	rs7231553	29075862	0.968	0.972
1131	rs7231163	29075875	0.968	0.972
1132	rs7231288	29075877	0.968	0.972
1133	rs7236324	29075905	0.969	0.974
1134	rs7235272	29076049	0.974	0.979
1135	rs73953901	29076052	0.975	0.979
1136	rs72479854	29076164	0.975	0.98
1137	rs73414280	29076223	0.975	0.98
1138	rs9961910	29076427	0.996	0.997
1139	rs9961925	29076490	0.953	0.991
1140	rs9961920	29076502	0.996	0.998
1141	rs9962423	29076794	0.953	0.99
1142	rs9962333	29076829	0.973	0.991
1143	rs2704038	29101612	0.966	0.989
1144	rs7506013	29103294	0.95	0.991
1145	rs2704039	29104055	0.957	0.991
1146	rs2848664	29104264	0.958	0.992
1147	rs3737378	29104564	0.951	0.993
1148	rs62095194	29104878	0.956	0.995
1149	rs4471764	29105881	0.965	0.999
1150	rs2704041	29106342	0.978	0.995

1151	rs62095195	29106821	0.968	0.996
1152	rs62095196	29106898	0.972	0.999
1153	rs62095222	29107614	0.971	0.996
1154	rs2704042	29107730	0.983	0.996
1155	rs2096918	29108087	0.98	0.999
1156	rs3108921	29108401	0.989	0.998
1157	rs3132910	29108505	0.977	0.996
1158	rs62095223	29108576	0.981	0.995
1159	rs1941938	29109036	0.98	0.997
1160	rs16962066	29109135	0.976	0.996
1161	rs2053825	29114149	0.974	0.991
1162	rs2053826	29114211	0.983	0.992
1163	rs7244098	29114496	0.973	0.991
1164	rs2053827	29114552	0.976	0.989
1165	rs4470222	29114621	0.978	0.989
1166	rs2595364	29115910	0.975	0.991
1170	rs1791176	29117509	0.972	0.991
1172	rs1791174	29119258	0.953	0.984
1173	rs1791173	29119357	0.972	0.993
1180	rs1626261	29121896	0.953	0.984
1185	rs1047433	29128572	0.979	0.997
1186	rs1791233	29128951	0.974	0.996
1187	rs57305209	29136013	0.953	0.971
1188	rs1667223	29136399	0.964	0.981
1191	rs7235277	29176873	0.978	0.996

1195	rs1791229	29182352	0.973	0.995
1196	rs1791201	29185268	0.962	0.995
1197	rs1791199	29187741	0.986	0.998
1198	rs1791198	29187889	0.975	0.996
1199	rs1667279	29218873	0.998	0.999
1200	rs3794878	29219289	0.971	0.997
1201	rs1791221	29219891	0.955	0.999
1202	rs9950458	29220600	0.971	0.994
1203	rs1667281	29220756	0.953	1
1204	rs6506935	29222804	0.966	0.993
1205	rs1078998	29228416	0.969	0.986
1206	rs12962843	29229632	0.958	0.981
1207	rs1551006	29233245	0.951	0.969
1208	rs1667288	29235544	0.951	0.976
1209	rs1667290	29236174	0.957	0.976
1210	rs4799589	29236256	0.974	0.986
1211	rs55817554	29237099	0.953	0.986
1212	rs1449086	29237765	0.968	0.98
1213	rs2848683	29238422	0.957	0.993
1214	rs1791183	29238686	0.958	0.994
1215	rs2595370	29239527	0.961	0.993
1216	rs1791172	29242412	0.987	0.99
1217	rs12970031	29248791	0.957	0.974
1218	rs1791175	29249515	0.958	0.967
1219	rs12327203	29259412	0.975	0.99

1220	rs1449082	29264028	0.984	0.993
1221	rs12605321	29268292	0.97	0.999
1222	rs11081707	29269624	0.965	0.994
1223	rs11664177	29273418	0.958	0.98
1224	rs34530749	29274349	0.954	0.975
1225	rs11660271	29275987	0.953	0.972
1226	rs1667295	29285892	0.982	0.982
1227	rs8092533	29289593	0.963	0.966
1228	rs12326689	29292118	0.965	0.966
1229	rs2220035	29329270	0.957	0.994
1230	rs4799317	29330269	0.978	0.997
1232	rs7228202	29333771	0.98	0.996
1233	rs7233679	29334151	0.981	0.996
1234	rs8088850	29334529	0.977	0.994
1235	rs4799602	29335201	0.976	0.994
1236	rs7233839	29350609	0.952	0.98
1237	rs17718318	29353154	0.979	0.99
1239	rs544671	29371713	0.979	0.971
1240	rs571431	29372347	0.961	0.968
1241	rs637765	29374301	0.969	0.98
1242	rs565800	29376236	0.993	0.997
1243	rs1786765	29378220	0.986	0.995
1244	rs668681	29378563	0.973	0.993
1245	rs11081716	29378568	0.965	0.997
1246	rs12051917	29379446	0.963	0.996

1247	rs7229200	29379465	0.958	0.985
1248	rs1842782	29379671	0.959	0.993
1249	rs1842784	29379887	0.961	0.996
1250	rs503172	29380294	0.977	0.999
1251	rs660653	29380713	0.994	0.998
1252	rs663297	29381313	0.993	0.998
1253	rs663803	29381442	0.992	0.997
1254	rs675881	29381838	0.981	0.999
1255	rs78186881	29382112	0.97	0.985
1256	rs567225	29382702	0.978	0.998
1257	rs1813019	29382789	0.955	0.997
1258	rs4799606	29383277	0.956	0.997
1259	rs150784627	29383823	0.956	0.996
1260	rs62095501	29383889	0.952	0.993
1261	rs139575087	29384237	0.967	0.983
1262	rs117944846	29384582	0.962	0.991
1263	rs138311296	29384925	0.955	0.994
1264	rs143035650	29384950	0.955	0.994
1265	rs145952287	29384959	0.955	0.994
1266	rs1680645	29385094	0.967	0.987
1267	rs186390648	29385108	0.965	0.995
1268	rs190600059	29385110	0.957	0.994
1269	rs686124	29385973	0.968	0.988
1270	rs116200618	29386007	0.96	0.993
1271	rs11876983	29386346	0.961	0.988

1272	rs11873806	29386352	0.96	0.988
1273	18:29386538	29386538	0.972	0.988
1274	rs150329568	29386730	0.971	0.996
1275	rs137997842	29386786	0.959	0.989
1276	rs141329483	29387232	0.974	0.996
1277	rs149526421	29387266	0.954	0.974
1278	rs35270696	29387413	0.951	0.981
1279	rs668996	29387420	0.959	0.981
1280	rs4799607	29388132	0.976	0.995
1281	rs61568697	29388798	0.979	0.996
1282	rs146378597	29390765	0.983	0.996
1283	rs11873449	29390999	0.985	0.996
1284	rs11873579	29391278	0.973	0.99
1285	rs76138476	29391474	0.986	0.997
1286	rs80262892	29392162	0.989	0.997
1287	rs78603984	29392305	0.99	0.997
1288	rs28451927	29392592	0.986	0.995
1289	18:29392956	29392956	0.99	0.997
1290	18:29392972	29392972	0.988	0.996
1291	rs148805916	29393343	0.99	0.996
1292	rs143721466	29395333	0.997	0.999
1293	rs11874924	29396140	0.987	0.991
1294	rs8084869	29402070	0.964	0.976
1295	rs8094542	29404095	0.977	0.983
1296	rs113287444	29404452	0.952	0.986

1297	rs12965893	29404891	0.977	0.983
1299	rs11081717	29406059	0.963	0.987
1300	rs111702464	29408313	0.962	0.993
1302	rs1815822	29408595	0.996	0.999
1303	rs111562873	29408627	0.959	0.987
1305	rs12327141	29410479	0.995	0.998
1307	18:29413964	29413964	0.959	0.985
1308	18:29413977	29413977	0.97	0.998
1310	rs28760310	29415830	0.974	0.995
1311	rs56046214	29416001	0.974	0.985
1312	rs12956638	29416575	0.998	0.998
1313	rs1786764	29416776	0.975	0.999
1314	rs12957830	29416825	0.996	0.998
1316	rs7235871	29417375	0.972	0.995
1317	rs10468902	29417515	0.996	0.998
1320	rs12607786	29418177	0.968	0.995
1321	rs76611163	29418590	0.972	0.998
1322	rs7226615	29419462	0.983	0.992
1324	rs16962510	29419959	0.973	0.998
1325	rs2035418	29421169	0.993	0.997
1329	rs11660557	29422498	0.969	0.995
1330	rs10153421	29422659	0.992	0.998
1332	rs12605636	29423569	0.97	0.995
1335	rs8085869	29424988	0.959	0.985
1337	rs12604640	29425755	0.989	0.997

1338	rs34846731	29426552	0.987	0.997
1344	rs112148461	29428676	0.979	0.998
1347	rs11876025	29429062	0.982	0.995
1349	rs4513172	29430466	0.979	0.997
1353	rs58098304	29431088	0.989	0.995
1356	rs7239098	29432278	0.976	0.997
1357	rs11081719	29432304	0.972	0.995
1358	rs7239629	29432332	0.99	0.995
1359	rs3737373	29432833	0.99	0.995
1361	rs3925536	29433323	0.99	0.995
1362	rs77349840	29434036	0.959	0.987
1363	rs55706254	29434727	0.99	0.995
1364	rs56303347	29434788	0.991	0.996
1365	rs112385937	29434969	0.986	0.998
1367	rs12608051	29436116	0.991	0.995
1368	rs111250816	29436305	0.986	0.996
1369	rs62095507	29436481	0.973	0.994
1370	rs80078064	29436596	0.985	0.997
1371	rs3810001	29437400	0.996	1
1373	rs113286310	29438926	0.987	0.995
1376	rs112629964	29443439	0.987	0.996
1377	rs28654480	29443964	0.956	0.992
1378	rs16962527	29446644	0.986	0.994
1379	rs113208588	29449984	0.996	0.997
1380	rs16962530	29451024	0.996	0.997

1381	rs16962533	29451312	0.992	0.994
1382	rs114921389	29453810	0.967	0.985
1383	rs16962539	29454837	0.976	0.987
1384	rs75328836	29456039	0.97	0.984
1385	rs12954849	29534152	0.971	0.992
1387	rs12607039	29535158	0.998	0.999
1389	rs12965971	29536762	0.995	0.997
1391	rs8098465	29537141	0.975	0.992
1392	rs4344833	29537659	0.974	0.991
1393	rs2421178	29537733	0.973	0.991
1395	rs9950155	29539196	0.971	0.989
1397	rs9950809	29539524	0.968	0.985
1398	rs4254394	29539540	0.992	0.993
1399	18:29539918	29539918	0.99	0.994
1400	rs144687892	29540099	0.99	0.992
1401	18:29540291	29540291	0.995	0.996
1403	rs146764168	29541038	0.961	0.983
1406	rs8097835	29542707	0.968	0.985
1407	rs8098106	29542839	0.993	0.992
1408	rs6506952	29543270	0.989	0.988
1409	rs7504671	29543754	0.963	0.979
1411	rs9949272	29544062	0.962	0.978
1412	rs2162378	29544191	0.961	0.978
1413	rs67236500	29544310	0.981	0.98
1415	rs12455103	29544839	0.978	0.976

1417	rs7244936	29546206	0.974	0.977
1418	rs8083160	29546666	0.952	0.972
1419	rs1030403	29546728	0.975	0.978
1420	rs4799611	29547027	0.984	0.988
1422	rs112318002	29548391	0.971	0.989
1423	rs8096737	29549163	0.988	0.992
1425	rs8097869	29549757	0.988	0.993
1427	rs974124	29550500	0.99	0.993
1428	rs12606043	29551384	0.988	0.996
1429	rs972358	29551829	0.995	0.999
1430	rs76868469	29551867	0.982	0.997
1431	rs7504605	29551974	0.993	0.999
1433	rs28562612	29552880	0.991	0.998
1434	rs8091659	29553229	0.985	0.993
1435	rs8091295	29553511	0.972	0.996
1436	rs4395150	29553629	0.987	0.996
1437	rs8095233	29553934	0.972	0.996
1438	rs7227644	29555525	0.978	0.992
1439	rs34954724	29555785	0.954	0.999
1440	rs9945434	29555806	0.99	0.99
1441	rs71361361	29556184	0.951	0.999
1442	rs4799614	29556725	0.966	0.984
1445	rs8093328	29566001	0.967	0.985
1446	rs10853417	29566087	0.959	0.976
1447	rs11081736	29566321	0.952	0.975

1448	rs7228224	29620320	0.951	0.981
1449	rs2433377	29621579	0.958	0.984
1450	rs2432697	29621645	0.975	0.993
1451	rs2433378	29621719	0.988	0.998
1452	rs336276	29621951	0.997	0.999
1453	rs336275	29622687	0.99	0.997
1454	rs336274	29624131	0.962	0.983
1455	rs1984583	29625376	0.958	0.989
1456	rs73954980	29629879	0.991	0.998
1457	rs10502580	29629956	0.993	0.998
1458	rs61149598	29630319	0.993	0.997
1459	rs55673393	29630335	0.987	0.994
1460	rs60790636	29630377	0.991	0.996
1461	rs75200655	29630597	0.989	0.993
1462	rs78074213	29630720	0.99	0.995
1463	rs79743274	29630981	0.955	0.975
1464	rs113978308	29631495	0.984	0.989
1465	rs142516845	29631872	0.982	0.987
1466	rs79440469	29632090	0.98	0.985
1467	rs57172349	29632410	0.968	0.981
1468	rs57264191	29632572	0.978	0.983
1469	rs61038301	29632749	0.977	0.982
1470	rs57807662	29632906	0.977	0.982
1471	rs59338045	29633051	0.975	0.981
1472	rs61628852	29633371	0.971	0.978

1473	18:29633441	29633441	0.97	0.977
1474	rs56403688	29633473	0.969	0.976
1475	rs60933882	29633592	0.968	0.975
1476	rs11874448	29633881	0.964	0.97
1477	rs61499404	29634517	0.955	0.961
1478	rs59939125	29634523	0.954	0.961
1479	rs59424188	29634545	0.955	0.961
1480	rs60448569	29634553	0.954	0.96
1481	rs8086733	29647869	0.972	0.98
1482	rs12604454	29661676	0.952	0.992
1483	18:29664057	29664057	0.99	0.997
1484	rs146019272	29664087	0.993	0.998
1485	rs4799325	29665712	0.952	0.99
1486	rs4799628	29666127	0.984	0.993
1487	rs8095867	29672559	0.967	0.981
1488	rs4799630	29674569	0.993	0.997
1489	rs4799631	29677865	0.98	0.991
1490	rs9953463	29679059	0.979	0.991
1491	rs61271315	29679560	0.987	0.999
1492	rs8085407	29682453	0.986	0.995
1495	rs28378426	29687904	0.984	0.999
1504	rs67929870	29727658	0.99	0.993
1505	rs9964960	29728387	0.996	0.998
1506	rs7240197	29728575	0.984	0.993
1507	rs9944759	29728939	0.991	0.995

1508	rs9944778	29728990	0.99	0.995
1509	rs11081750	29728999	0.968	0.988
1510	rs9955753	29729284	0.983	0.99
1511	rs62095576	29729302	0.983	0.99
1512	rs62095577	29729314	0.983	0.989
1513	rs62095578	29729473	0.986	0.992
1514	rs62095579	29729634	0.984	0.991
1515	rs72639475	29729848	0.981	0.99
1516	rs12457134	29730058	0.975	0.989
1517	rs12457138	29730096	0.974	0.989
1518	rs9959235	29730317	0.973	0.988
1519	rs58425259	29731724	0.993	0.993
1520	rs7232533	29731937	0.963	0.979
1521	rs62095587	29732642	0.962	0.981
1522	rs8092667	29745316	0.962	0.981
1524	rs7233250	29748206	0.975	0.991
1525	rs8093507	29749194	0.957	0.982
1527	rs9963287	29754787	0.978	0.991
1528	rs9954856	29755144	0.956	0.984
1529	rs11873351	29757070	0.982	0.991
1530	rs7233927	29778804	0.992	0.994
1531	rs16962815	29779003	0.994	0.996
1532	rs16962818	29779094	0.995	0.998
1533	rs1561815	29779487	0.996	0.999
1534	18:29779623	29779623	0.983	0.988

1535	18:29779627	29779627	0.982	0.986
1536	18:29779631	29779631	0.993	0.997
1537	rs9953120	29779760	0.979	0.998
1538	rs9675713	29779845	0.973	0.994
1539	rs9955488	29779976	0.964	0.993
1540	rs9955566	29780022	0.963	0.997
1541	rs8098463	29780275	0.967	0.996
1542	rs8098766	29780548	0.968	0.996
1543	rs9967163	29780768	0.973	0.996
1544	rs8095196	29781337	0.97	0.996
1545	rs8095362	29781488	0.971	0.996
1546	rs8096290	29781521	0.971	0.996
1547	rs8095388	29781645	0.969	0.997
1548	rs28451536	29781704	0.973	0.995
1549	rs11874395	29781875	0.974	0.996
1550	rs28410852	29781966	0.975	0.996
1551	rs7226471	29782404	0.976	0.996
1552	rs4799330	29783478	0.986	0.992
1553	rs9948943	29783885	0.978	0.993
1555	rs6506962	29785472	0.992	0.998
1556	rs9955257	29785697	0.976	0.996
1557	rs9945892	29786094	0.989	0.997
1560	rs8085389	29786676	0.99	0.997
1562	rs6506963	29786952	0.977	0.997
1563	rs6506964	29787102	0.983	0.998

1564	rs4799639	29787900	0.993	0.998
1565	rs28759083	29788357	0.983	0.996
1567	rs9952703	29788571	0.976	0.998
1568	rs6506965	29788783	0.995	0.999
1569	rs17663127	29789165	0.98	0.997
1570	rs59324074	29789212	0.965	0.996
1571	rs7244677	29789273	0.998	1
1572	rs35117504	29789330	0.974	0.996
1574	rs28667990	29789716	0.991	1
1575	rs7231916	29790029	0.998	0.997
1577	rs9959013	29790147	0.987	0.994
1578	rs9959058	29790376	0.996	0.995
1579	rs9959396	29790520	0.973	0.998
1580	rs9948580	29790740	0.97	0.993
1581	rs335519	29790889	0.995	0.993
1583	rs9960121	29791987	0.969	0.991
1584	rs7234401	29792796	0.97	0.998
1585	rs72939741	29792900	0.982	0.997
1586	rs335517	29793708	0.995	0.996
1587	rs9954412	29793984	0.964	0.997
1588	rs335516	29794067	0.993	1
1589	rs335515	29794247	0.996	1
1591	rs335514	29795743	0.989	0.993
1593	rs11876428	29796185	0.989	0.997
1594	rs9748763	29796188	0.992	0.99

1595	rs9961628	29796602	0.97	1
1596	rs9961748	29796763	0.993	0.998
1598	rs9953963	29797548	0.997	0.999
1599	rs112703073	29797790	0.965	0.996
1600	rs9945189	29798540	0.971	0.999
1601	rs16962856	29799240	0.971	0.999
1602	rs10502585	29799313	0.998	0.999
1603	rs28690950	29799379	0.999	1
1604	rs673408	29800119	0.998	0.999
1605	rs11873961	29800550	0.997	0.998
1609	rs114809532	29801578	0.971	0.989
1610	rs1596166	29801762	0.997	0.998
1611	rs662158	29802450	0.993	0.997
1612	rs28439453	29803042	0.967	0.995
1613	rs620982	29803271	0.996	0.998
1616	rs28605495	29804465	0.966	0.995
1617	rs28874686	29804633	0.966	0.994
1619	rs654488	29804863	0.994	0.997
1620	rs72939680	29804906	0.979	0.998
1621	rs9950258	29805134	0.995	0.997
1623	rs641476	29805408	0.996	0.998
1624	rs115782251	29806412	0.967	0.988
1625	rs60370195	29806577	0.984	0.989
1626	rs72939689	29806686	0.977	0.997
1627	rs9956980	29807475	0.965	0.995

1628	rs628064	29807966	0.985	0.999
1629	rs9959940	29808188	0.994	0.996
1630	rs9960051	29808253	0.987	0.987
1631	18:29808286	29808286	0.994	0.996
1632	18:29810168	29810168	0.964	0.994
1633	18:29810406	29810406	0.972	0.994
1634	18:29810459	29810459	0.976	0.99
1635	18:29810685	29810685	0.965	0.988
1636	18:29810799	29810799	0.961	0.995
1637	18:29810806	29810806	0.976	0.989
1638	rs8088129	29811400	0.985	0.999
1639	rs142897005	29811642	0.968	0.993
1640	18:29811775	29811775	0.972	0.997
1641	18:29811919	29811919	0.964	0.995
1643	18:29812288	29812288	0.964	0.995
1646	18:29812616	29812616	0.971	0.988
1647	rs146838716	29812691	0.962	0.983
1648	18:29812847	29812847	0.995	0.998
1649	18:29812861	29812861	0.987	0.998
1650	18:29812880	29812880	0.951	0.975
1651	18:29812899	29812899	0.964	0.996
1652	18:29813202	29813202	0.995	0.998
1653	18:29813332	29813332	0.992	0.997
1654	18:29813465	29813465	0.972	0.98
1655	rs143096454	29813483	0.971	0.994

1656	rs148213939	29813490	0.991	0.995
1657	rs146987023	29813654	0.962	0.996
1658	rs138076853	29813664	0.966	0.988
1659	rs143735687	29813704	0.961	0.994
1661	rs11877942	29813749	0.973	0.996
1662	rs11872463	29813908	0.976	0.999
1664	rs16962878	29815039	0.977	1
1665	rs11873446	29815359	0.952	0.976
1666	rs9952884	29816126	0.959	0.992
1667	rs666950	29816345	0.968	0.994
1668	rs11081752	29816693	0.974	0.999
1669	rs670059	29817015	0.963	0.992
1670	rs11081753	29817088	0.964	0.997
1671	rs16962889	29817275	0.963	0.995
1672	rs141320655	29819271	0.956	0.975
1673	rs28516907	29819290	0.963	0.993
1674	rs16962893	29819553	0.998	0.999
1675	rs9965650	29820012	0.997	0.997
1676	rs9951980	29821496	0.996	0.997
1677	rs9964636	29821823	0.959	0.989
1678	rs116683083	29822860	0.956	0.985
1681	rs28482447	29835384	0.952	0.98
1682	rs73419859	29837135	0.957	0.98
1683	rs636809	29837990	0.973	0.991
1684	rs647499	29839065	0.964	0.988

1685	rs9950965	29840562	0.958	0.982
1686	rs682459	29840940	0.965	0.999
1687	rs16962918	29841532	0.958	0.987
1688	rs11661174	29842149	0.976	0.993
1689	rs114766416	29842577	0.955	0.993
1690	rs16962924	29843312	0.977	0.993
1691	rs9510	29843771	0.987	0.993
1692	rs635901	29844062	0.99	0.995
1693	rs9953127	29846619	0.979	0.998
1694	rs11665544	29852229	0.979	0.996
1695	rs58018218	29852587	0.962	0.994
1696	rs8086990	29853287	0.975	0.993
1697	rs645526	29853357	0.978	0.988
1698	rs8087394	29853498	0.976	0.994
1699	rs8086611	29853504	0.98	0.997
1700	rs115940538	29853623	0.964	0.995
1701	rs59106127	29855904	0.967	0.996
1702	rs668567	29859715	0.956	0.994
1703	rs16962965	29860726	0.99	0.998
1704	rs9947150	29862100	0.991	0.998
1705	rs618862	29862459	0.974	0.995
1706	rs11660076	29862874	0.951	0.972
1707	rs16962971	29865203	0.988	0.994
1708	rs9960751	29866522	0.993	0.998
1709	rs16962977	29867504	0.956	0.985

1711	rs79610002	29869024	0.981	0.988
1712	rs606576	29870550	0.969	0.982
1713	rs59756419	29871179	0.974	0.992
1714	rs9916917	29871293	0.97	0.978
1715	rs140436689	29872199	0.976	0.98
1716	rs625863	29873113	0.982	0.985
1717	rs624130	29873469	0.995	0.996
1718	rs596653	29874152	0.982	0.985
1719	rs1614242	29876374	0.957	0.968
1720	rs613941	29879188	0.954	0.961
1721	rs613616	29879203	0.952	0.959
1722	rs188596899	29879342	0.962	0.959
1723	rs192834776	29884140	0.955	0.953
1724	rs625361	29890411	0.959	0.996
1725	rs628733	29890448	0.958	0.992
1726	rs682827	29890456	0.957	0.992
1727	rs677137	29890888	0.959	0.993
1728	rs583065	29891593	0.952	0.999
1729	rs671090	29892355	0.966	0.994
1730	rs58863502	29892360	0.962	0.991
1731	rs57546420	29892583	0.962	0.991
1733	rs606638	29894992	0.981	0.997
1734	rs688824	29895258	0.977	0.995
1735	rs35221772	29895406	0.98	0.993
1737	rs660031	29895971	0.986	0.997

1739	rs681347	29896375	0.985	0.997
1740	rs644274	29897165	0.993	0.999
1742	rs687850	29897276	0.991	0.998
1743	rs643836	29897282	0.991	0.998
1744	rs657479	29897346	0.991	0.998
1745	rs632368	29897489	0.995	0.999
1746	rs656946	29897513	0.995	0.997
1747	rs672867	29898615	0.999	1
1749	rs736218	29899067	0.976	0.998
1750	rs638142	29899143	0.983	0.998
1751	rs614014	29899316	0.998	0.999
1752	rs11872811	29899564	0.992	0.995
1754	rs642914	29900377	0.989	0.995
1756	rs667604	29900697	0.965	0.998
1762	rs75653391	29905293	0.968	0.995
1764	rs679614	29905598	0.959	0.999
1765	rs668857	29905667	0.984	0.996
1767	rs1841119	29905935	0.973	0.994
1771	rs594344	29908095	0.97	0.992
1774	rs677721	29910144	0.979	0.99
1777	rs7230250	29911099	0.959	0.987
1780	rs1689030	29912990	0.97	0.981
1781	rs73956883	29913974	0.977	0.989
1782	rs7239218	29916143	0.994	0.997
1783	rs16963135	29918072	0.989	0.994

1784	rs55642668	29918995	0.995	0.998
1786	rs7240587	29920215	0.999	1
1787	rs7240755	29920280	0.996	0.999
1788	rs148011543	29921302	0.995	0.997
1789	rs140756273	29921310	0.974	0.986
1791	rs638868	29922921	0.974	0.99
1792	rs7227973	29922937	0.977	0.995
1793	rs7228337	29923155	0.988	0.991
1794	rs16963169	29923219	0.993	0.994
1796	rs79418481	29923478	0.991	0.994
1797	rs113569763	29923529	0.989	0.992
1798	rs10502588	29924034	0.991	0.993
1799	rs73956895	29924125	0.983	0.989
1800	rs10502589	29924170	0.99	0.99
1801	rs144868925	29924978	0.988	0.992
1802	rs75853118	29925057	0.985	0.992
1807	rs16963196	29928766	0.995	0.996
1808	rs59676860	29929031	0.995	0.996
1809	rs968099	29929220	0.962	0.984
1810	rs10502590	29931146	0.995	0.998
1811	rs7243964	29931688	0.961	0.984
1812	rs16963218	29931734	0.997	0.999
1813	rs16963225	29932387	0.996	0.998
1814	rs16963230	29932541	0.984	0.992
1815	rs57613491	29932916	0.995	0.997

1816	rs16963239	29934728	0.994	0.996
1818	rs16963245	29936245	0.995	0.997
1819	rs11876773	29937076	0.956	0.981
1820	rs11877702	29938043	0.991	0.993
1821	rs73956901	29938608	0.992	0.995
1822	rs73956902	29938839	0.99	0.994
1823	rs902951	29939277	0.992	0.995
1825	rs902950	29939451	0.992	0.995
1826	rs2421455	29939659	0.991	0.995
1827	rs57478295	29940334	0.991	0.995
1828	rs73958403	29943016	0.99	0.995
1829	rs73958404	29943699	0.984	0.989
1830	rs73958405	29943796	0.991	0.995
1831	rs4485446	29943917	0.989	0.993
1832	rs55866517	29944186	0.986	0.991
1833	rs56945883	29944733	0.982	0.992
1836	rs4499310	29947372	0.995	0.996
1837	rs11663076	29948616	0.975	0.993
1838	rs16963270	29949519	0.97	0.995
1839	rs16963272	29949599	0.995	0.997
1841	rs16963275	29950516	0.997	0.998
1842	rs16963279	29950624	0.995	0.996
1844	rs1458860	29952104	0.998	0.999
1848	rs73958407	29953066	0.98	0.999
1849	rs76531138	29953099	0.98	0.999

1853	rs41527551	29956215	0.975	0.998
1854	rs2034968	29957035	0.977	0.998
1856	rs35779665	29957236	0.993	0.994
1858	rs1964517	29957789	0.978	0.997
1862	rs8097263	29961185	0.986	0.998
1863	rs59456493	29961419	0.979	0.997
1865	rs73958410	29961852	0.98	0.997
1867	rs73958411	29963663	0.976	0.991
1868	rs16963285	29963697	0.98	0.998
1872	rs16963289	29967673	0.993	0.999
1873	rs11665190	29968263	0.991	0.998
1874	rs11665147	29968307	0.993	0.999
1875	rs11665150	29968336	0.993	0.999
1876	rs56808255	29969877	0.987	0.992
1878	rs11873325	29970176	0.988	0.992
1879	rs72931888	29970204	0.969	0.985
1880	rs10502596	29971458	0.988	0.994
1881	rs10048362	29971934	0.993	1
1882	rs16963314	29972397	0.974	0.998
1883	rs3826611	29972565	0.99	1
1884	rs3786310	29972724	0.984	0.998
1885	rs13381344	29973731	0.979	0.995
1886	rs75169444	29974337	0.96	0.994
1887	rs7233422	29975867	0.975	0.993
1888	rs150540621	29976050	0.975	0.989

1889	rs149298513	29976117	0.967	0.998
1890	rs4561543	29976832	0.97	0.992
1891	rs146861316	29976875	0.966	0.998
1892	rs60577454	29977097	0.973	0.989
1893	rs58503085	29977138	0.973	0.989
1894	rs113981046	29977207	0.962	0.996
1895	rs111972357	29977212	0.962	0.996
1896	rs112588530	29977333	0.965	0.997
1897	rs4597393	29977395	0.968	0.992
1898	rs113660695	29977486	0.965	0.998
1899	rs146256688	29977602	0.965	0.998
1900	rs142696115	29977811	0.972	0.999
1901	rs11504782	29978241	0.971	0.999
1902	rs11504783	29978281	0.964	0.998
1903	rs11504784	29978378	0.961	0.998
1904	rs150098544	29979575	0.959	0.99
1905	rs142582020	29980015	0.963	0.997
1906	rs184329528	29980443	0.969	0.988
1907	rs150623139	29980950	0.962	0.998
1908	rs75662342	29981018	0.961	0.993
1909	rs147489502	29981089	0.967	0.996
1910	rs72931898	29981526	0.968	0.988
1911	rs73958413	29981687	0.967	0.996
1912	rs73958414	29981975	0.959	0.999
1913	rs113937997	29982472	0.958	0.999

1914	rs16963334	29984571	0.951	1
1915	rs73958415	29984660	0.95	1
1916	rs59211646	29986271	0.951	1
1917	rs16963339	29986488	0.966	0.989
1918	rs73958418	29987767	0.965	0.996
1919	rs72933507	29990368	0.954	0.98
1920	rs58220828	29990755	0.96	0.998
1921	rs8085402	29992418	0.963	0.989
1922	18:29992655	29992655	0.962	0.996
1923	rs112269952	29995306	0.974	0.997
1924	rs57978863	30004181	0.96	0.988
1925	rs10438935	30005111	0.975	0.993
1926	rs1458858	30005527	0.977	0.995
1928	rs7243481	30007288	0.98	0.995
1929	rs7243086	30007292	0.98	0.995
1931	rs73958422	30009211	0.968	0.984
1932	rs968177	30009261	0.985	1
1933	rs11081762	30010410	0.97	0.992
1934	rs11081763	30010524	0.985	0.996
1935	rs11081764	30010533	0.985	0.996
1936	rs7228553	30010986	0.952	0.999
1937	rs7229026	30011272	0.99	1
1938	rs10468833	30011496	0.991	1
1939	rs10775451	30011745	0.991	1
1940	rs72933528	30012002	0.981	0.998

1941	rs10853421	30012160	0.992	1
1942	rs72933532	30013620	0.995	1
1943	rs60928334	30013818	0.995	1
1944	rs55641647	30014430	0.981	0.999
1945	rs55730180	30014584	0.987	0.996
1946	rs73954805	30014682	0.982	0.999
1947	rs73954806	30014975	0.99	0.998
1948	rs73954807	30015014	0.99	0.998
1949	rs16959889	30015464	0.984	0.999
1950	rs16963360	30015479	0.989	0.998
1951	rs11081765	30016039	0.996	1
1952	rs13381184	30016502	0.978	1
1953	rs8093474	30016646	0.953	0.998
1954	rs16963365	30016695	0.984	0.998
1955	rs8093807	30016880	0.992	1
1956	rs72933534	30016897	0.977	0.999
1957	rs8096329	30016980	0.991	1
1958	rs72933545	30017226	0.99	1
1959	rs72933548	30018382	0.96	0.995
1960	rs13380968	30018384	0.978	0.992
1961	rs2055060	30018417	0.98	0.994
1962	rs2052757	30019340	0.966	0.998
1963	rs72933552	30019903	0.953	0.999
1964	rs59630704	30020252	0.977	0.993
1965	rs8091089	30020985	0.967	0.996

1966	rs9807749	30022518	0.974	0.996
1967	rs16963373	30023069	0.958	0.997
1968	rs8087581	30024150	0.958	0.997
1969	rs17744064	30024828	0.979	0.998
1970	rs17811690	30026629	0.965	1
1971	rs13381541	30027100	0.959	0.995
1972	rs13380952	30027101	0.959	0.995
1973	rs12604457	30027337	0.968	0.998
1975	rs115304339	30028484	0.953	0.994
1976	rs17811714	30028554	0.991	0.998
1978	rs1350551	30028974	0.988	0.992
1979	rs1863605	30029033	0.992	0.997
1980	rs8094711	30029193	0.974	0.996
1981	rs61273573	30030411	0.962	1
1982	rs17744164	30030412	0.971	0.997
1983	rs61311884	30030417	0.99	0.998
1984	rs17811798	30030682	0.987	0.995
1985	rs55941037	30030732	0.986	0.994
1986	rs729693	30031637	0.978	0.98
1987	rs729692	30031755	0.977	0.979
1988	rs73954814	30032275	0.968	0.972
1989	rs1492830	30041994	0.958	0.987
1990	rs1492832	30042099	0.959	0.987
1991	rs9950386	30042753	0.963	0.987
1992	rs9967126	30044178	0.985	0.992

1993	rs66569206	30044365	0.987	0.992
1994	rs72935565	30044465	0.99	0.994
1995	rs11872611	30057400	0.995	0.998
1996	rs12956654	30057622	0.97	0.991
1997	rs61157243	30058388	0.987	0.993
1998	rs11660611	30058647	0.965	0.988
1999	rs11664107	30058655	0.951	0.99
2000	rs16963405	30058825	0.994	0.998
2001	rs11874256	30059271	0.992	0.998
2002	rs12604259	30060634	0.982	0.995
2003	rs55924842	30060745	0.974	0.993
2004	rs12606310	30060796	0.981	0.995
2005	rs11081770	30061514	0.979	0.995
2006	rs12604199	30062038	0.977	0.996
2007	rs56192440	30062458	0.976	0.996
2008	rs16963407	30063476	0.975	0.996
2009	rs17454053	30064200	0.973	0.997
2010	rs61444191	30064338	0.973	0.997
2011	rs56383034	30064754	0.972	0.998
2012	rs56367129	30064842	0.972	0.998
2013	18:30067156	30067156	0.97	0.998
2014	rs12607349	30067468	0.97	0.998
2015	rs10502604	30067645	0.97	0.998
2016	rs6506970	30068363	0.969	0.997
2017	rs17736978	30069176	0.951	0.995

2018	rs17736990	30069710	0.95	0.995
2019	rs72937430	30069799	0.95	0.996
2020	rs2131357	30071032	0.95	0.997
2021	rs60971244	30072039	0.969	0.99
2022	rs66847257	30075319	0.985	0.982
2023	rs7242416	30077009	0.962	0.953
2025	rs12954406	30083269	0.982	0.997
2026	rs12454779	30084085	0.973	0.988
2027	rs12962995	30084919	0.958	0.982
2028	rs7226840	30090754	0.988	0.998
2029	rs9953890	30091078	0.987	0.999
2030	rs9954414	30091635	0.971	0.997
2031	rs2015877	30091940	0.971	0.987
2032	rs2015883	30092058	0.96	0.981
2033	rs1985293	30092327	0.974	0.989
2034	rs6506971	30107893	0.983	0.991
2035	rs8094262	30110426	0.973	0.985
2036	rs1549517	30111037	0.966	0.98
2037	rs12457401	30112885	0.97	0.986
2038	rs8098481	30114732	0.97	0.989
2039	rs59709499	30118402	0.959	0.964
2040	rs16963530	30122686	0.994	0.998
2041	rs72941789	30122740	0.993	0.998
2042	rs16963537	30124311	0.977	0.986
2043	rs12150800	30124494	0.977	0.986

2044	rs4799649	30126466	0.965	0.973
2045	rs28374729	30138732	0.968	0.982
2046	rs11663345	30142189	0.99	0.995
2047	rs167940	30144623	0.962	0.981
2048	rs179387	30144842	0.985	0.998
2049	rs11659767	30145492	0.993	0.997
2050	rs56145763	30148024	0.972	0.985
2051	rs330296	30148051	0.966	0.985
2052	rs179352	30148923	0.969	0.979
2053	rs8087017	30148924	0.952	0.99
2054	rs1693320	30149209	0.969	0.979
2055	rs191319	30149229	0.968	0.979
2056	rs186664	30149831	0.962	0.974
2057	rs162336	30150269	0.966	0.976
2058	rs330294	30150946	0.984	0.984
2059	rs330293	30151276	0.97	0.987
2060	rs330292	30152146	0.982	0.982
2061	rs330291	30152458	0.98	0.98
2062	rs330290	30152473	0.978	0.978
2063	rs330288	30153219	0.979	0.98
2064	rs330287	30153467	0.976	0.975
2065	rs330286	30153736	0.969	0.987
2066	rs8083035	30156973	0.976	0.994
2067	rs8083282	30157009	0.976	0.994
2068	rs1648668	30158043	0.996	1

2069	rs1705470	30158047	0.972	0.988
2070	rs330298	30158089	0.996	1
2071	rs58245863	30158110	0.973	0.994
2072	rs162418	30158256	0.97	0.988
2073	rs162419	30158289	0.97	0.988
2074	rs162420	30158396	0.994	0.999
2075	rs330299	30158490	0.967	0.987
2076	rs16963576	30158536	0.95	0.983
2077	rs7239008	30159103	0.953	0.984
2078	rs162421	30159128	0.973	0.987
2079	rs162422	30159167	0.973	0.987
2080	rs330300	30159586	0.988	0.998
2081	rs7238961	30159613	0.952	0.984
2082	rs330301	30159882	0.984	0.996
2083	rs330302	30160240	0.988	0.997
2084	rs330303	30160793	0.99	0.997
2085	rs330304	30160922	0.99	0.997
2086	rs330307	30161792	0.991	0.997
2087	rs330308	30161982	0.99	0.997
2088	rs937400	30163377	0.983	0.992
2089	rs937401	30163383	0.989	0.995
2090	rs937402	30163532	0.987	0.993
2091	rs1693310	30164014	0.997	0.997
2092	rs1705477	30164324	0.995	0.996
2093	rs28446378	30164552	0.951	0.985

2094	18:30164848	30164848	0.987	0.991
2095	rs1693311	30165317	0.999	0.999
2096	18:30166325	30166325	0.988	0.99
2097	rs12606513	30166329	0.987	0.99
2098	rs67841603	30166522	0.997	0.999
2099	rs66506014	30166778	0.993	0.996
2100	rs66841150	30167043	0.994	0.998
2101	rs66849639	30167052	0.993	0.998
2102	rs1693313	30167136	0.992	0.998
2103	rs1693314	30167164	0.992	0.998
2104	rs1693315	30167269	0.991	0.997
2105	rs1705451	30167286	0.971	0.993
2106	rs12960149	30167325	0.99	0.997
2107	rs12960182	30167376	0.99	0.997
2108	rs1705450	30167616	0.988	0.996
2109	rs1693316	30168166	0.983	0.994
2110	rs1705449	30168203	0.981	0.993
2111	rs1705448	30168204	0.981	0.993
2112	rs2666805	30168587	0.954	0.999
2113	rs1705446	30169380	0.978	0.993
2114	rs1693317	30171188	0.973	0.993
2115	rs1693318	30171203	0.972	0.993
2116	rs1705445	30171724	0.955	0.992
2117	rs1705444	30171765	0.971	0.993
2118	rs1705443	30172407	0.968	0.992

2119	rs1705441	30173027	0.966	0.992
2120	rs1705440	30173034	0.966	0.992
2121	rs1693319	30173579	0.963	0.991
2122	rs189796	30174635	0.963	0.991

Table S2. Details of the association analysis between Val122Ile and traits related to medical history. Beta values are reported as effect size for linear regression models. Odds Ratios are reported as effect size for logistic regression models.

Trait	Regression Model	Effect	P
Health Status	Linear	-0.12	0.1624
High Blood Pressure	Logistic	1.09	0.629
Migraine headaches	Logistic	0.76	0.3044
Brain injury or concussion	Logistic	0.54	0.09202
Been unconscious for longer than 5 min	Logistic	0.75	0.2828
Epilepsy or have had a seizure	Logistic	0.92	0.822
Meningitis or encephalitis	Logistic	0.51	0.5055
Stroke	Logistic	0.60	0.4818
Heart disease	Logistic	1.55	0.2005
Liver disease	Logistic	0.55	0.06978
Thyroid disease	Logistic	1.46	0.3463
Asthma	Logistic	0.75	0.1748
Diabetes	Logistic	1.03	0.9164
Cancer	Logistic	1.01	0.9849
Hospitalizations N	Linear	0.00	0.9816
Hospitalizations ≥ 10	Logistic	1.04	0.9337
Outpatient surgeries > 0	Logistic	0.76	0.1055
Outpatient surgeries ≥ 10	Logistic	6.87	7.81×10^{-5}
Emergency room visits N	Linear	-0.09	0.5493
Emergency room visits ≥ 10	Logistic	0.85	0.6344
Prescription medications for nerves	Logistic	0.85	0.5059
Prescription medications for sleep	Logistic	0.79	0.2811
Prescription medications for depression	Logistic	1.04	0.8562
Prescription medications for headaches	Logistic	1.01	0.9699

Prescription medications for energy	Logistic	1.16	0.7743
Prescription medications containing steroids	Logistic	0.44	0.02752

Table S3. LD blocks identified in the genomic regions investigated in the haplotype association analysis (GRCh37/hg19 chr 18: 28,171,770–30,174,635). The rsId of the variants are reported in Table S1.

LD Block	Variants (Order ID)
#1	2 3 4 5 6 7 8 9 10 11 12 13 14 15 16 17 18 19 20 21 22 23 24 25 26 27 28 29 30 31
#2	32 33 34 35 36 37 38 39 40 41 42 43 44 45 46 47 48 49 50 51
#3	52 53 54 55 56 57
#4	58 59
#5	60 61
#6	62 63 64 65 66 67 68 69 70 71 72 73 74 75 76 77 78 79 80 81 82 83 84 85 86 87 88 89 90 91 92 93 94
#7	95 96 97 98 99 100 101 102 103 104 105 106 107 108 109 110 111 112 113 114 115 116 117 118 119 120
#8	121 122 123 124 125 126 127
#9	128 129 130 131 132 133 134 135 136 137 138 139 140
#10	141 142 143 144 145 146 147 148 149 150 151 152 153 154 155
#11	156 157 158 159 160 161 162 163 164 165 166
#12	169 170 171 172 173 174 175 176 177 178 179 180 181 182 183 184 186 187 188 189
#13	190 191 192 193 194 195 196 197 198 199 200 201
#14	202 203 204 205 206 207 208 209 210 211 212 213 214
#15	215 216 217 218 219 220
#16	221 222 223 224 225 226 227 228 229 230 231 232 233 234 235 236 237 238 239
#17	242 243 244 245 246 247 248 249 251 252
#18	255 256 257 258 259 261 262 263 264 265 266 269 278 283 284 287 291 292 293 301 303 306
#19	315 316 317 318 320 321 322 323 324 326 327 328 329 330 331 332 333 334 335 336 340 342 351 359 360 362 363 366

#20	374 375 377 378 379 380 381 382 384 385 386 388 389 390 392 395 396
#21	399 402 403
#22	405 406 409
#23	412 414 415 416 417 418 419 420 421 422 423 424 426
#24	427 428 429
#25	430 431 433 434
#26	438 439 441 442 443
#27	444 445 446 447 448 449 450 451 452 453 454 455 456 457 458 459 460 461 462 463 464 465 466 467 468 469 470 471 472 473 474 475 476 477 478 479 480 481 482 483 484 485 486 487 488 489
#28	490 491 492 493 494 495 496 497 498 499 500 502
#29	503 504 505 506 507 508 509 510 511 512 513 514
#30	515 516 517 518 519 520 521 522 523 524 525 526 527 528 529
#31	530 531 532 533 534 535 536 537 538 539 540 541 542 543 544 545 546 547 548 549 550 551 552 553 554 555 556 557 558 559 560 561 563 564 565 566 567 568 569 570 572
#32	573 574 575 576 577 578 579 580 581 582 583 584 585 586 587
#33	588 589 590 591
#34	593 594 595 597 598 599 600
#35	603 604 606 607 608 609 610 611 612 613 614 615 617 618 619 620 621 623 625 626 627 628 629 630 631 632 633 634 635 636
#36	638 639
#37	641 642 643
#38	644 645 646 647 648 649 650 651 652 653 654 655 657 658 659 660 662 663 664 665 666 667 668 669 670 672 673 674 675
#39	677 678 679 680 681 682
#40	684 686 689 690 691 692 694 698 699 700 701 702 704 705 706 707 708 709 710 711 712 713 714 715 716 717 718 720 721 723 724 726 727 728 729 730 732 733 734 735 737 738 739 740 741 742 743 744 746 747 748 749 750 751 752 753 754 755 757 758 759 760 761 763 764 765 766 767 768 769 771 772 773 774 775

#41	776 777 780 781 782 783 784 785 786 788 789 791 793 795 796 798 801 802 804 806 807 808 809 810 811 812 813 814 815 816 817 818 819 820 821 822 823 825 826 827 828 829 830 831 833 834 835 836 837 838 839 840 841 842 843 844 845 846 847 848 849 850 851 852 853 854 855 856 857 858 859 860 861 862 863 864 865 866 867 868 869 870 871 872
#42	873 874 875
#43	878 879 880 881 882 883
#44	885 886 887 888 889 890 891 892 893 894 895
#45	896 897 898 900
#46	902 903 904 905 906 907 908 909 910 911 912 913 914 915 916 917 918 919 920 922 923 924 925 926 927 928 929 930 931 932 933 934 935
#47	937 938
#48	940 941 942 943 944 945 947 948 949 950 951 952 954 956 957 959 960
#49	970 972 973
#50	974 976 978 979 980 981 982 983
#51	984 985 986 987 988 989 990 991 992 993 994 995 996 997 998 999 1000 1001 1002 1003 1004 1005 1006 1007 1008 1009 1010 1011 1012 1013 1014 1015 1016 1017 1018 1019 1020 1023 1024 1025
#52	1026 1027 1028 1030 1031 1032 1033 1034 1035 1036 1038 1040 1041 1042 1043 1044
#53	1046 1047 1048
#54	1050 1051 1052 1053 1054 1055 1056 1057 1058 1059 1060 1061 1062 1063 1064 1065 1066 1067 1068 1069 1070 1071 1072 1073 1074 1075 1076 1077 1078 1079 1080 1081 1082 1083 1084 1085 1086 1087 1088 1089 1090 1091 1092
#55	1093 1094 1095 1096 1097 1098 1099 1100 1101 1102 1103 1104 1105 1106 1107 1108 1109 1110 1111
#56	1112 1113 1114 1115 1116
#57	1118 1119 1120
#58	1121 1122 1123 1124 1125 1126 1127 1128 1129 1130 1131 1132 1133 1134 1135 1136 1137 1138 1139 1140 1141 1142
#59	1144 1145 1146 1147 1148 1149
#60	1151 1152 1153 1154 1155 1156 1157 1158 1159 1160 1161 1162 1163 1164 1165
#61	1166 1170 1172 1173 1180 1185 1186

#62	1195 1196 1197
#63	1199 1200 1201 1202 1203 1204 1205 1206
#64	1208 1209 1210 1211 1212 1213 1214 1215 1216
#65	1217 1218 1219 1220 1221 1222 1223 1224 1225
#66	1227 1228
#67	1230 1232 1233 1234 1235 1236 1237
#68	1239 1240 1241 1242 1243 1244 1245 1246 1247
#69	1248 1249
#70	1250 1251 1252 1253 1254 1255 1256
#71	1257 1258 1259 1260 1261 1262 1263 1264 1265 1266 1267 1268 1269 1270 1271 1272 1273 1274 1275 1276 1277 1278 1279 1280 1281 1282 1283 1284 1285 1286 1287 1288 1289 1290 1291 1292 1293
#72	1294 1295
#73	1300 1302 1303 1305 1307 1308 1310 1311 1312 1313 1314 1316 1317 1320 1321 1322 1324 1325 1329 1330 1332 1335 1337 1338 1344 1347 1349 1353 1356 1357 1358 1359 1361 1362 1363 1364 1365 1367 1368 1369 1370 1371 1373 1376 1377 1378 1379 1380 1381 1382 1383 1384
#74	1387 1389 1391 1392 1393 1395 1397 1398 1399 1400 1401 1403 1406 1407 1408 1409 1411 1412 1413 1415 1417 1418 1419 1420 1422 1423 1425
#75	1427 1428 1429 1430 1431 1433 1434 1435 1436 1437 1438 1439 1440 1441 1442
#76	1445 1446
#77	1448 1449 1450 1451 1452 1453 1454 1455
#78	1456 1457 1458 1459 1460 1461 1462 1463 1464 1465 1466 1467 1468 1469 1470 1471 1472 1473 1474 1475 1476 1477 1478 1479 1480
#79	1482 1483 1484
#80	1485 1486 1487
#81	1488 1489 1490 1491 1492 1495 1504 1505 1506 1507 1508 1509 1510 1511 1512 1513 1514 1515 1516 1517 1518 1519
#82	1520 1521
#83	1525 1527 1528 1529

#84	1530 1531 1532 1533 1534 1535 1536 1537
#85	1538 1539 1540 1541 1542 1543 1544 1545 1546 1547 1548 1549 1550 1551 1552 1553 1555 1556 1557 1560 1562 1563 1564 1565 1567 1568 1569 1570 1571 1572 1574 1575 1577 1578 1579 1580 1581 1583
#86	1586 1587 1588 1589 1591
#87	1593 1594 1595 1596 1598 1599 1600 1601 1602 1603 1604 1605 1609 1610 1611 1612 1613 1616 1617 1619 1620 1621 1623 1624 1625 1626 1627 1628 1629 1630 1631 1632 1633 1634 1635 1636 1637 1638 1639 1640 1641 1643 1646 1647 1648 1649 1650 1651 1652 1653 1654 1655 1656 1657 1658 1659 1661 1662 1664 1665 1666 1667 1668 1669 1670 1671 1672 1673 1674 1675 1676 1677 1678
#88	1681 1682 1683 1684 1685 1686 1687 1688 1689 1690 1691
#89	1693 1694 1695 1696 1697 1698 1699 1700 1701 1702 1703 1704 1705 1706 1707 1708 1709 1711 1712 1713 1714
#90	1715 1716 1717 1718 1719 1720 1721 1722 1723
#91	1724 1725 1726 1727 1728 1729 1730 1731 1733 1734 1735 1737 1739 1740 1742 1743 1744 1745 1746 1747
#92	1750 1751
#93	1754 1756 1762 1764 1765 1767
#94	1771 1774
#95	1777 1780 1781 1782 1783 1784 1786 1787 1788 1789 1791 1792 1793 1794 1796 1797 1798 1799 1800 1801 1802 1807 1808 1809 1810 1811 1812 1813 1814 1815 1816 1818 1819 1820 1821 1822 1823 1825 1826 1827 1828 1829 1830 1831 1832 1833 1836 1837 1838 1839 1841 1842 1844 1848 1849 1853 1854 1856 1858 1862
#96	1863 1865
#97	1867 1868 1872 1873 1874 1875 1876 1878 1879 1880 1881 1882 1883 1884 1885 1886 1887 1888 1889 1890 1891 1892 1893 1894 1895 1896 1897 1898 1899 1900 1901 1902 1903 1904 1905 1906 1907 1908 1909 1910 1911 1912 1913 1914 1915 1916 1917 1918 1919 1920 1921 1922 1923 1924
#98	1925 1926 1928 1929 1931 1932 1933 1934 1935 1936 1937 1938 1939 1940 1941 1942 1943 1944 1945 1946 1947 1948 1949 1950 1951 1952 1953 1954 1955 1956 1957 1958 1959 1960 1961 1962 1963 1964 1965
#99	1966 1967 1968 1969 1970 1971 1972 1973 1975 1976 1978 1979 1980 1981 1982 1983 1984 1985 1986 1987 1988
#100	1989 1990 1991 1992 1993 1994

#101	1995 1996 1997 1998 1999 2000 2001 2002 2003 2004 2005 2006 2007 2008 2009 2010 2011 2012 2013 2014 2015 2016 2017 2018 2019 2020 2021
#102	2022 2023
#103	2025 2026 2027
#104	2028 2029 2030 2031 2032 2033
#105	2034 2035 2036
#106	2037 2038
#107	2039 2040 2041 2042 2043 2044
#108	2045 2046 2047 2048 2049 2050 2051 2052 2053 2054 2055 2056 2057 2058 2059 2060 2061 2062 2063 2064 2065 2066 2067
#109	2068 2069 2070 2071 2072 2073 2074 2075 2076 2077
#110	2078 2079
#111	2080 2081 2082 2083 2084 2085 2086 2087 2088 2089 2090 2091 2092 2093 2094 2095 2096 2097 2098 2099 2100 2101 2102 2103 2104 2105 2106 2107 2108 2109 2110 2111 2112 2113 2114 2115 2116 2117 2118 2119 2120 2121 2122

Table S4. Association results of all haplotypes tested with respect to "having 10 or more outpatient surgeries" among Val122Ile carriers.

Haplotype	Case,Control Frequencies	Chi Square	P Value
Block 1			
CCACTACAGCCCCGCCGCGCGCGAACATCA	0.500, 0.414	0.237	0.6265
CCACTACAGCCTATTCATGGATATATTTCC	0.375, 0.297	0.224	0.6358
CCGCTACAGCCCCGCCGCGCGCGAACATCA	0.125, 0.126	0	0.99
TTATCGTGATTCAGTTATACGCGTGTTCTC	0.000, 0.125	1.139	0.286
CCACTACAGCCCCTTCATGGATATATTTCC	0.000, 0.014	0.11	0.7407
Block 2			
ACCGAGTAAATTGCGTCAGG	0.625, 0.557	0.144	0.7041
GGATTACGGCCCATCCTGAA	0.250, 0.297	0.084	0.7725
GGCGAGTAAATTGCCCTGAA	0.125, 0.098	0.064	0.8003
GGATTATGGCCCATCCTGAA	0.000, 0.017	0.137	0.7109
GGATTACGGACCATCCTGAA	0.000, 0.014	0.11	0.7407
Block 3			
GGCTCG	0.500, 0.456	0.061	0.8056
AACACG	0.000, 0.252	2.677	0.1018
AATATA	0.250, 0.199	0.125	0.724
AACTCG	0.250, 0.058	4.814	0.0282
GACTCG	0.000, 0.026	0.214	0.6437
Block 4			
GG	0.500, 0.557	0.104	0.747
AA	0.500, 0.405	0.289	0.5911
GA	0.000, 0.037	0.308	0.5786

Block 5			
AT	0.875, 0.608	2.344	0.1258
CC	0.125, 0.345	1.677	0.1954
CT	0.000, 0.047	0.397	0.5288
Block 6			
TCCGCCTTTTGGGTAGCATGCAGCCGCCAGCTG	0.500, 0.304	1.403	0.2362
CTTTTGTCGCATACGAGACTCGCGTCGTGATCA	0.125, 0.262	0.767	0.3812
TCCTCCTTTTGGGTAGCATGCAGCCGCCAGCTG	0.000, 0.098	0.867	0.3518
TTCTCGTCGCATACAAGACTCGCGTCCTGATCA	0.000, 0.087	0.763	0.3825
TTTTTGGCGCATACAAGGCTTGCGTCGTGATCA	0.000, 0.064	0.549	0.4586
TTTTTGTCGCATACGAGACTCGCGTCGTGATCA	0.250, 0.048	6.246	0.0125
TTTTTGGCGCATACAAGGCTTGCGTCGTGACTG	0.000, 0.035	0.294	0.588
TTTGCCTCGCATACAAGACTCGCGTCGTGATCA	0.125, 0.014	5.98	0.0145
CCTTTGTCGCATACGAGACTCGCGTCGTGATCA	0.000, 0.014	0.111	0.7392
Block 7			
TATAACGCGTAGTTGTGGCACTGCGC	0.375, 0.476	0.321	0.571
AGAGCTATACGACCAGAAAGTACAAA	0.250, 0.341	0.289	0.5907
AGTGCTATATGACCAGAAAGTTCAAA	0.000, 0.088	0.768	0.3807
AGTACCACATGGCTAGGGAATTCAAC	0.125, 0.061	0.548	0.4592
AGAGCTATACGACCAGAACGTAGCGC	0.125, 0.014	5.985	0.0144
AGAGCTATATGACCAGAAAGTACAAA	0.125, 0.010	7.915	0.0049
Block 8			
TGAGAGA	0.375, 0.355	0.014	0.9059
CAGGATC	0.250, 0.338	0.27	0.6036
TGAAATC	0.125, 0.158	0.065	0.7987

CGGGGTC	0.000, 0.068	0.579	0.4469
TGAGATC	0.000, 0.044	0.371	0.5423
CGGGATC	0.250, 0.024	13.9	2.00E-04
Block 9			
ATGTTAAGACCCT	0.500, 0.416	0.228	0.6327
TCAGGCCCTTAAA	0.375, 0.334	0.057	0.8106
ATGTTACGACCCA	0.000, 0.152	1.428	0.2322
TTATGACGTTCAA	0.125, 0.064	0.469	0.4936
Block 10			
ACGATAGCCGTCTAA	0.375, 0.405	0.03	0.8627
ACGACAACCGTCTAA	0.125, 0.395	2.396	0.1217
TTTTTGGTAACGATT	0.000, 0.095	0.834	0.3613
TTTTTGGTAACCATT	0.375, 0.051	14.709	1.00E-04
ACGATAGTCGTCTAA	0.000, 0.020	0.165	0.6842
ACTACAACCGTCTAA	0.000, 0.017	0.137	0.7109
Block 11			
AATAGTTTCAG	0.000, 0.324	3.792	0.0515
CACAGTCTTGT	0.375, 0.307	0.167	0.6824
CACAGTTCTAG	0.125, 0.145	0.026	0.8723
CTCGACCTTGT	0.000, 0.091	0.799	0.3713
CACAGCCTTGT	0.375, 0.051	14.653	1.00E-04
CACAGTTCCAG	0.125, 0.034	1.858	0.1728

CACAGTTTCAG	0.000, 0.017	0.137	0.7109
Block 12			
CCCATATGCGTAAGACCCTC	0.000, 0.331	3.907	0.0481
GCACCGTATGGAGGGACTCC	0.375, 0.262	0.507	0.4763
CCCACATGCAGTGAGCGCTT	0.125, 0.139	0.012	0.913
GTACCGCATGGAGGGACCTC	0.000, 0.084	0.736	0.3909
GCACCGTATGGAGGGACTTC	0.000, 0.055	0.466	0.4949
GCACCGTATGGAGGGACCTC	0.375, 0.030	24.395	7.85E-07
CCACCGTATGGAGGGACTTC	0.125, 0.031	2.179	0.1399
CCCATATGCGGAAGACCCTC	0.000, 0.017	0.137	0.7109
GCCCCGTATGGAGGGACTCC	0.000, 0.015	0.119	0.7299
Block 13			
AATCCGGTAAGC	0.375, 0.659	2.762	0.0965
GGCTTAACGTCT	0.375, 0.199	1.481	0.2237
AATTCGGTAAGC	0.250, 0.132	0.933	0.334
Block 14			
CGTGTGTAGGGTT	0.375, 0.634	2.231	0.1353
TCAAACCGATACC	0.250, 0.222	0.034	0.8535
TCAGTGTAGGGTT	0.125, 0.076	0.269	0.6043
TCAAACTAGGGTT	0.000, 0.020	0.166	0.6833
TCTGTGTAGGGCC	0.250, 0.007	35.494	2.56E-09
Block 15			
GACAAG	0.625, 0.797	1.408	0.2355

ATTGGA	0.125, 0.196	0.251	0.6166
GACAGA	0.250, 0.007	35.494	2.56E-09
Block 16			
CTTGTTCATACAGGCTATT	0.500, 0.716	1.77	0.1833
TAAACCACCGTACATCTGC	0.000, 0.108	0.967	0.3255
TTTGTCCCTACAGGCCTTT	0.500, 0.061	22.381	2.24E-06
TAAACCACCGTGCATCTGC	0.000, 0.071	0.61	0.4349
CTTGTTCATACAGGCCTTT	0.000, 0.017	0.137	0.7109
Block 17			
TGTTTGCGAT	0.500, 0.537	0.043	0.8352
TGTGCAAGGT	0.250, 0.213	0.064	0.8003
CCCTCGCAAC	0.250, 0.162	0.437	0.5084
TGCTCGCGAT	0.000, 0.054	0.456	0.4993
CCCTCGCGAT	0.000, 0.030	0.251	0.6166
Block 18			
AGGAGCTATACTGTATTACACC	0.750, 0.301	7.32	0.0068
TAAGACAACCTCGATCATGATT	0.125, 0.253	0.685	0.408
TAAGACATCCTCAATCATGATT	0.000, 0.125	1.139	0.286
AGAGGGTATCCTGTACTACACT	0.000, 0.111	1	0.3172
AGAGGGTATCCTGAACTACGCT	0.125, 0.074	0.286	0.5928
TAAGACAACCTCAATCATGATT	0.000, 0.054	0.456	0.4993
AGAGGGTATCCTGAACATCACT	0.000, 0.034	0.279	0.5971
AGGAGCTATCCTGTATTACACC	0.000, 0.030	0.251	0.6166

AGAGGGTATCCTGTATTACACC	0.000, 0.014	0.11	0.7407
Block 19			
TAAACATCACCATAATGGTAGCGTTGGG	0.750, 0.324	6.327	0.0119
TAGTCATCACCATAATGACGGCGGTAGG	0.125, 0.210	0.339	0.5606
TCAACATCACCATAATGACGGCGGTAAG	0.000, 0.098	0.864	0.3527
TAGTCATCACCATAATGACGGCGGAAGG	0.000, 0.078	0.673	0.4121
TCAACATCACCAAAATGACGGCGGTAGG	0.125, 0.071	0.333	0.5637
AAAACATCACCGTAATGGTGGCATTGGC	0.000, 0.074	0.641	0.4233
TAGATCCTGTTATGGCCGTGAAGTTGGG	0.000, 0.061	0.517	0.472
TAAACATCACCATAATGGTGGCGTTGGG	0.000, 0.037	0.309	0.5786
TAGTCATCACCATAATGACGGCGTTGGG	0.000, 0.027	0.222	0.6374
Block 20			
TAAATCTGGTACTTGGC	0.750, 0.355	5.25	0.022
CGATTTTCAGTCTTGAT	0.125, 0.294	1.081	0.2984
CGATTTTCAGTCTTGAC	0.000, 0.084	0.736	0.391
CATATCTGGTATCACGC	0.000, 0.081	0.704	0.4013
CGATTTCCAGTCTTGAT	0.125, 0.074	0.287	0.5924
CAAAACTGGTATCAGGC	0.000, 0.074	0.641	0.4233
CAATTTTCAGTCTTGAC	0.000, 0.037	0.308	0.5786
Block 21			
GAT	0.875, 0.649	1.765	0.184
GTC	0.125, 0.270	0.841	0.3591
ATC	0.000, 0.081	0.704	0.4014
Block 22			
CCC	0.750, 0.696	0.108	0.7426

GTG	0.125, 0.172	0.123	0.7259
GTC	0.000, 0.074	0.641	0.4234
CTC	0.125, 0.057	0.638	0.4243
Block 23			
GTTTTGAGGCCAA	0.250, 0.291	0.062	0.803
GATCTAATACCGA	0.125, 0.196	0.25	0.6168
GTATGAATACCAA	0.250, 0.189	0.187	0.6655
GTTTGACTACCAA	0.125, 0.074	0.286	0.5928
ATTTGAATATTAG	0.000, 0.074	0.641	0.4234
GATCTAATACCAA	0.000, 0.071	0.608	0.4354
GTTTGAATACTAA	0.000, 0.047	0.397	0.5288
GTTTTAATGCCAA	0.000, 0.017	0.136	0.712
GTTTTAATACCAA	0.000, 0.014	0.111	0.7394
GAACTAATACCGA	0.250, 0.007	35.344	2.76E-09
GTTCTAATACCAA	0.000, 0.014	0.11	0.7407
Block 24			
GAT	0.250, 0.378	0.548	0.4592
GGC	0.250, 0.284	0.044	0.8342
AAT	0.500, 0.196	4.436	0.0352
GGT	0.000, 0.142	1.317	0.2511
Block 25			
CCTG	0.500, 0.649	0.751	0.386
TCTG	0.500, 0.274	1.981	0.1592
TTCA	0.000, 0.071	0.61	0.4349

Block 26			
GCGGT	0.875, 0.638	1.902	0.1678
GCAGT	0.125, 0.226	0.46	0.4979
ATGTG	0.000, 0.084	0.735	0.3914
GTGTG	0.000, 0.034	0.281	0.5962
Block 27			
GACTCGCCAACTATTAAAAGGCGACAACTCGTGGTGGGTGAGCCCC	0.250, 0.358	0.397	0.5286
AGGACGTTGGTCGCCGCATCGGGAAGACCCGTGCCGGGTCAGCTCC	0.000, 0.234	2.428	0.1192
AGGACGTTGGTCGCCGCATCGGGAAGATCCGTGCCGAGTCAGCTTT	0.125, 0.115	0.008	0.9294
AGGAAATTGGTCGCCGCGTCAGAGAGGCCGACTCCCGAACGCTCTT	0.375, 0.091	7.053	0.0079
AGGACGCCAACTATTAAATCGGGAAAACCCGTGGTGGGTGAGCCCC	0.125, 0.061	0.548	0.4592
AGGACGTTGGTCGCCGCATCGGGAAGACCCGTGCCGGGTCAGCCCC	0.000, 0.033	0.269	0.6038
AGGAAATTGGTCGCCGCGTCGGGGAGGCCGACGCCGGAACAGCCCC	0.000, 0.030	0.251	0.6166
AGGACGTTGGTCGCCGCATCGGGGAGGCCCGTGCCGGGTCAGCTCC	0.000, 0.020	0.165	0.6842
GGGACGTTGGTCGCCGCATCGGGAAGACCCGTGCCGGGTCAGCTCC	0.000, 0.016	0.128	0.7204
AGGACGTTGGTCGCCGCATCGGGAAGACCCGTGGTGGGTGAGCCCC	0.000, 0.014	0.11	0.7407
Block 28			
CTAGGGCGGAGG	0.250, 0.368	0.47	0.4931
CTAGGGCGGAAC	0.000, 0.338	4.028	0.0448
ACTAAATCTAAC	0.125, 0.115	0.008	0.9294
CCTGGGCCTCAC	0.375, 0.095	6.688	0.0097
CTAGGGCGGAAG	0.125, 0.078	0.24	0.6245
Block 29			
TCGTTTTGGGCT	0.625, 0.568	0.105	0.7462
CTACAAAACATC	0.375, 0.375	0	1

CTACAAAACGTC	0.000, 0.044	0.367	0.5446
Block 30			
CGATTCGGATGTAAG	0.496, 0.509	0.005	0.9441
TAGGCTTCGCACGGC	0.125, 0.166	0.093	0.7602
TAGGCTTGATGCGGC	0.125, 0.084	0.164	0.6858
TAGGCTTGATGTAAG	0.121, 0.073	0.264	0.6076
TGGGCTTGATGTAAG	0.000, 0.041	0.338	0.5612
CGATTCTGATGTAAG	0.129, 0.035	1.889	0.1693
TAGGCCGGATGTAAG	0.000, 0.037	0.308	0.5786
TAGGCTGGATGTAAG	0.004, 0.032	0.203	0.6522
CAGGCTTGATGTAAG	0.000, 0.024	0.194	0.6599
Block 31			
CCTCTGACCGTTAGCGTTCGTCAACAGTCTTATCTCGACCC	0.250, 0.267	0.011	0.9146
TTCACTTTTCCCGCTTCCGAATTGAGTCTATCCTGGAGCTG	0.125, 0.166	0.093	0.7599
CCTCTGACCGTTAGCGTTCGTCAACAGTCTTCTCTCGACTC	0.125, 0.125	0	0.9997
CCTCTGACCGTTAGCGTTCGTCAACAGTCTCCCTGGAGATG	0.250, 0.108	1.589	0.2074
CCTCTGACCGTTAGCGTTCGTCAACAGTCTCCCTGGAGCTG	0.000, 0.088	0.772	0.3797
TTCACTTTTCCCGCTTCCGAATTGAATCTATCCTGGAGCTG	0.125, 0.081	0.199	0.6557
TTCACTTTTCTTAGCGTTCGTCAGAAGCTATCCTGCGGCTG	0.125, 0.078	0.24	0.6245
CCTCTGACCGTTAGCGTTCGTCAACAGTCTTCTTGCAACTG	0.000, 0.037	0.309	0.5786
Block 32			
TGCACTAGGGTTGAC	0.250, 0.311	0.135	0.7134
CTCGTCGAGACGAGT	0.250, 0.267	0.011	0.9151
CTCGTCAAGACGAGT	0.250, 0.203	0.107	0.7432
CTTACTAGAGCTGAC	0.250, 0.111	1.467	0.2258

CTTACTAGGGCTGAC	0.000, 0.037	0.308	0.5786
TGCACTAGGGCTGAC	0.000, 0.034	0.279	0.5971
CTCGTTAAGACGAGT	0.000, 0.017	0.137	0.7109
Block 33			
CCTT	0.375, 0.446	0.159	0.6903
TTCC	0.250, 0.392	0.66	0.4164
CTCC	0.250, 0.155	0.524	0.4691
Block 34			
GCAATAA	0.250, 0.318	0.165	0.685
GCGTATC	0.125, 0.209	0.338	0.5609
GCATATC	0.250, 0.203	0.107	0.7432
TTAAAAA	0.250, 0.104	1.72	0.1897
GCAAAAA	0.125, 0.094	0.087	0.7678
TCAAAAA	0.000, 0.038	0.313	0.5755
GTAAAAA	0.000, 0.017	0.142	0.7061
GCATAAA	0.000, 0.017	0.137	0.7109
Block 35			
GTACTGCTCCGGCAGAAGAATGAACAGTTG	0.250, 0.324	0.197	0.6572
ATGGTATCTTATTTTGGAGGAAGCTTACCA	0.000, 0.196	1.937	0.164
ATACTGCCTTATTTTGGAGGAAAACAGTTG	0.000, 0.139	1.281	0.2577
GTACAGCTCCGGCAGAAGAGAGAACAGTTG	0.125, 0.115	0.008	0.9294
GCACAGCTCCGTCAGAAGAGAGAACAGTTG	0.250, 0.101	1.828	0.1764
ATACTGCTCCGGCAGAAGAATGAACAGTTG	0.125, 0.057	0.638	0.4243
ATAGTGCCTTATTTTGGAGGAAAACAGTTG	0.125, 0.017	4.705	0.0301
GTACAGCTCCGTCAGAAGAGAGAACTGTTG	0.000, 0.017	0.137	0.7109

Block 36			
GT	0.625, 0.659	0.04	0.8425
TG	0.250, 0.284	0.044	0.8342
GG	0.125, 0.057	0.638	0.4243
Block 37			
CTA	0.875, 0.706	1.081	0.2985
TAG	0.125, 0.284	0.975	0.3234
Block 38			
TGCCATCTTGTGTGTAAAGGTTCGTCTTG	0.625, 0.314	3.462	0.0628
TGCCATCTTGTGTGTAAAGGTTCGTCAGG	0.250, 0.182	0.238	0.6259
CATTGGTCCACAACCGGCACACGGTCTTG	0.125, 0.169	0.108	0.7428
CATTGGTCCACAACCGGCACACGAACTTA	0.000, 0.101	0.9	0.3429
TGCCATCTTGTGTGTAAAGGTTCGTGTTG	0.000, 0.081	0.704	0.4013
CGTCATCCTGCGTGCAAAGCTTCGTCTTG	0.000, 0.065	0.555	0.4561
TGCCGTCCTGCGTCCGGCACACGGTCTTG	0.000, 0.024	0.194	0.6599
CGTCGTCCTGCGTGCAAAGCTTCGTCTTG	0.000, 0.014	0.11	0.7406
Block 39			
TAGGGA	0.750, 0.581	0.916	0.3386
CCACTG	0.125, 0.179	0.156	0.693
CAGGGA	0.000, 0.149	1.39	0.2383
CCGCTG	0.000, 0.081	0.704	0.4014
Block 40			
CTACGCGGCAGGTGAACGTCACCGGTGTACATCCCTACACTGACTTGATGAGTTAACTGCAGGGTGGTTCACGGA	0.375, 0.270	0.431	0.5113

CTACGCGTGAAACGGGCGCCGGTAGCGCGCATTGTCGCGGTAGCTCAGTAATTAGGTTGTTAAGCGCTCCAGAAC	0.125, 0.115	0.008	0.9294
CTACGCGTGAGATGAGTTCCGGTGATGTGCATTGTCGAGGCAGTTTAGTAGTTAGGTTGTTAAGCGCTCCAGAAC	0.250, 0.101	1.828	0.1764
TTACGCGGCGAGTAAACGTAAGCGGTATACTTTCCTACACTGACGTGATGATTTAACTGTTGAACACGCTTGGGA	0.000, 0.101	0.9	0.3429
CCCGAGATGAGACGGGCGCCGGTAGCGCGTACTGTCGCGGTAGCTTAGCAATCAGGTTATTGAGCGCTCCAGAGC	0.000, 0.088	0.768	0.3807
CTACGCGTCAGATGAGTTCCGGTGATGTGCATTGTCGCGGCAGTTTAGTAGTTAGGTTGTTAAGCGCTCCAGAAC	0.000, 0.081	0.704	0.4014
CCCGAGATGAGACGGGCGCCGGTAGCGCGTACTGTCGCGGTAGCTTAGCAATCAGGTTGTTGAGCGCTCCAGAGC	0.125, 0.064	0.469	0.4936
CTACGCGTGAAACGGGCGCCGGTAGCGCGCATTGTCGCGGTAGCTCAGTAATTAGGTCGTTAAGCGCTCCAGAAC	0.000, 0.047	0.397	0.5288
CTACGCGGCAGGTGAACGTCAGCGGTGTACATTCCTACACTGACGTGATGATTTAACTGTTGAACACGCTAGGGA	0.000, 0.034	0.279	0.5971
CTACGCGGCAGGTGAACGTCGGCGGTGTGCATCCCTACACTGACTTGATGATTTGACTGTTGAGCGCTCCAGAGC	0.125, 0.010	7.835	0.0051
CCCGAGATGAGACGGGCGCCGGTAGCGCGCATTGTCGCGGTAGCTCAGTAATTAGGTTGTTAAGCGCTCCAGAAC	0.000, 0.014	0.11	0.7407
CTACGCGGCAAGTGAACGTCAGCGGTATACATTCCTACACTGACGTGATGATTTAACTGTTGAACACGCTAGGGA	0.000, 0.014	0.11	0.7407
Block 41			
TCTTTTCGACGATGTTGCTGGACAATGCCAATCTTAAGTCGGCGTTTGAGCTACGGGACGAAAGCCATCTCTCTGACTCCTGCC	0.375, 0.277	0.371	0.5424

TTTTCTCGGTTAGGATACTTCGTGACACTGACTGTAAGTCGGCGTTTGAGCTACGGGACGAAAGTTATCT CTCTGACTCGGCTA	0.125, 0.182	0.173	0.6771
TCTTTTCGACGTTGTTGCTGGACAATGCCAATTTTAAGTCGGTGTTTGAGCTACGGGACGAAAGCCATCTC TCTGACTCCTGCC	0.000, 0.152	1.428	0.2322
CTCCTCTGACGATCTTACCGGACATTGACAATTTGGGACTAACAAAAACCTCCTAAACTAGGGTCTGCAG TCTCTCTAGGGCTA	0.250, 0.111	1.467	0.2258
TTTTTTCAGTGAGGATACTTCGTGACACTGTCTGTAAGTCGGCGTTTGAGCTACGGGACGAAAGCTATCT CTCTGACTCCTGCC	0.250, 0.098	1.966	0.1609
CTCCTCTGACGATCTCATCGGACATTGACAATTTGGGACTAACAAAAACCTCCTAAACTAGGGTCCGCA GTCTCTCTAGGGCTA	0.000, 0.081	0.704	0.4014
TTTTTTCAGTGAGGATACTTCGTGACACTGACTGTAAGTCGGCGTTTGAGCTACGGGACGAAAGTTATCT CTCTGACTCCTGCC	0.000, 0.078	0.673	0.4122
Block 42			
GCC	0.375, 0.486	0.388	0.5335
GTC	0.375, 0.429	0.093	0.7604
CTG	0.250, 0.084	2.638	0.1044
Block 43			
CGGGGA	0.500, 0.361	0.645	0.422
CGGAGG	0.375, 0.324	0.091	0.7628
TGGGGA	0.125, 0.209	0.338	0.5609
CACATG	0.000, 0.105	0.933	0.3341
Block 44			
GACTCCCGTGT	0.500, 0.416	0.228	0.6327
GACTCCTGTGT	0.250, 0.277	0.028	0.8662
GGTACTCACAT	0.000, 0.122	1.104	0.2935

GGTATTCACAT	0.000, 0.101	0.9	0.3429
TACTCCCGTGC	0.125, 0.081	0.199	0.6555
Block 45			
TCGC	1.000, 0.777	2.278	0.1312
CTAG	0.000, 0.115	1.035	0.3091
TCGG	0.000, 0.074	0.641	0.4234
TTAG	0.000, 0.034	0.279	0.5971
Block 46			
TCCCGCTTACTCGCCTGCGTCCGAGTGTGATGT	0.500, 0.321	1.137	0.2862
TCCCGCTTACTCGCCTGCGTCCGGAGAGTGCAC	0.250, 0.233	0.012	0.9116
CAGTATCAGTTTACCTGCGTCCGGAGAGTGCAC	0.000, 0.101	0.9	0.3429
TCCCGCTTACACGTTCGTGCCTCAATGTGGTGT	0.125, 0.081	0.199	0.6554
TCCCGCTTACTCGCCTTCATTCGAGTGTGATGT	0.000, 0.068	0.579	0.4469
TCGCGCTTGCTCGCCTGCGTCCGGAGAGTGCAC	0.000, 0.068	0.578	0.447
TCCCGCTTATTTACCTGCGTCCGGAGAGTGCAC	0.125, 0.063	0.5	0.4797
CAGTATTTGTTTACCTGCGTCCGGAGAGTGCAC	0.000, 0.041	0.338	0.5612
Block 47			
CC	0.875, 0.841	0.067	0.796
CT	0.125, 0.081	0.199	0.6555
TT	0.000, 0.078	0.673	0.4122
Block 48			
CTCCGGGCAATTTTTTT	0.750, 0.510	1.802	0.1795
CCTCGGGCAATTTTTTT	0.250, 0.274	0.022	0.8822
TTCCAAATGGACGCCAC	0.000, 0.067	0.575	0.4482
CTCTGGGCAATTTTTTT	0.000, 0.065	0.552	0.4575

TTCCAAATGGACGTTTT	0.000, 0.031	0.254	0.6144
TTCCAAATGAACGTTTT	0.000, 0.030	0.248	0.6188
CCCCGGGCAATTTTTTT	0.000, 0.014	0.11	0.7407
Block 49			
CGC	0.625, 0.561	0.13	0.718
TGA	0.250, 0.301	0.095	0.7575
CAC	0.125, 0.139	0.012	0.913
Block 50			
AGGTGAGG	1.000, 0.926	0.641	0.4234
CTACCCAA	0.000, 0.061	0.517	0.4721
AGGTCAGG	0.000, 0.014	0.11	0.7407
Block 51			
AATAACATTGCTGCCGTTCGGGTAACTTTGACAGCCGTGG	0.500, 0.399	0.333	0.5639
GATAACAATGCTACCGTTCGGGCCGTCAACGTTCTTCCAA	0.250, 0.247	0	0.9826
GATAACAATGCTACCGTTCGGGCCGTCAACGTTGTCCTAG	0.125, 0.118	0.003	0.9535
GATAACAATGCCACCGTTCGAGCCGTCAACGTTCTTCCAA	0.125, 0.074	0.286	0.5928
GGCGCATACTTTATTACCGAGTCCGTCAACACAGTTCCAA	0.000, 0.061	0.517	0.4721
GATAACAATGCTACCGTTCGGGCCGTCAACGTTGTTCCAA	0.000, 0.034	0.279	0.5971
AATAACAATGCTGCCGTTCGGGTAACTTTCACAGCCGTAG	0.000, 0.030	0.251	0.6166
GATAACAATGCTACCGTTCGGGCCGTCAACACAGTTCCAA	0.000, 0.014	0.11	0.7407
Block 52			
TGCAATCTACTCGGTG	0.500, 0.395	0.362	0.5476
GAAGGATGTGGGGGTG	0.375, 0.341	0.04	0.8425
GAAGGATGTGGGAAAA	0.000, 0.074	0.641	0.4234
TACAGTCTACTGGGTG	0.000, 0.071	0.609	0.4352

TACAGTCTACTCGGTG	0.125, 0.051	0.859	0.3539
TGCAGTCTACTGGGTG	0.000, 0.026	0.217	0.6414
TAAAGATGTGGGGGTG	0.000, 0.020	0.165	0.6842
Block 53			
AAA	0.750, 0.767	0.012	0.9113
CTT	0.250, 0.230	0.018	0.8931
Block 54			
GCGTTTATGATAATGAAAAGTCCCTATTGGGTTGTCCAAATAT	0.125, 0.250	0.655	0.4184
ATACCCGCAGAGTTCGGGGGCCCGGGCCATACCAATCGGGAGT	0.125, 0.169	0.109	0.7414
ATACCCGCAGAGTTCGGGGGCCCGGGCCATACCAATTGGGAGC	0.125, 0.145	0.026	0.8723
GCACCCGCAGAGTCCGGGGACTCCTATTGGGTTGTCCAAATAT	0.125, 0.115	0.008	0.9293
ATACCCGCAGAGTTCGGGGGCCTGGGCCATACCAATCGGGAGT	0.125, 0.108	0.024	0.8771
GCACCCGCAGAGTTCGGGGGCCCGGGCCATACCAATCGGGAGT	0.125, 0.051	0.863	0.353
ATACCCGCAGAGTTCGGGGGCCCGGGCCATACCAACCAAATAT	0.250, 0.047	6.476	0.0109
ATACCCGCAGAGTTCGGGGGCCCGGGCCGTACCAATCGGGAGT	0.000, 0.030	0.251	0.6167
GCATTTATGATAATGAAAAGTCCCTATTGGGTTGTCCAAATAT	0.000, 0.017	0.137	0.7109
Block 55			
GAGTCGATAGTTGGAAGGA	0.375, 0.277	0.37	0.5429
AATTTAGCGACCAAGAAGA	0.125, 0.149	0.036	0.8499
AATTTAGCGACTAGGTGGG	0.125, 0.145	0.025	0.8743
GAGTCGGTAGTTGGAAGAA	0.125, 0.135	0.007	0.9337
ATTCTAGCGACTAGGAGGA	0.125, 0.122	0.001	0.9773
AATCTAGCGACTAGGTGGG	0.125, 0.078	0.239	0.6248
AATTCAGCGACTAGGTGGG	0.000, 0.037	0.309	0.5785
GAGTCGGTAGTTGGGTGGA	0.000, 0.020	0.165	0.6841

Block 56			
ACTTG	0.750, 0.723	0.028	0.866
GTCCA	0.250, 0.277	0.028	0.866
Block 57			
ATA	0.875, 0.855	0.026	0.8723
GCG	0.125, 0.142	0.018	0.8924
Block 58			
ACAATAAACAGTGGCCATTGCA	0.750, 0.703	0.084	0.7724
ACAATGGGTGACTATTGTTGCA	0.125, 0.110	0.018	0.8946
ACAATGGGTGACTATTGCTACC	0.125, 0.072	0.317	0.5736
GATGGGGGTGACTATTGCCAGC	0.000, 0.061	0.517	0.4721
GATGGGGGTGACTATTGCTACC	0.000, 0.036	0.297	0.586
Block 59			
ATCCGG	0.625, 0.537	0.242	0.6228
ACTCGG	0.375, 0.382	0.002	0.969
GCTAAA	0.000, 0.064	0.548	0.4592
ACTCGA	0.000, 0.014	0.11	0.7407
Block 60			
GTAGGGTAGAAGTCG	0.625, 0.524	0.321	0.5709
GTATGAAAAAAGTCG	0.125, 0.243	0.597	0.4398
GTAGGGTAGCCGCCG	0.250, 0.095	2.117	0.1457
AATGAGTGGACACTA	0.000, 0.064	0.548	0.4592
GTATGATAGAAGTCG	0.000, 0.054	0.456	0.4993
GTAGGGTAGACGCCG	0.000, 0.014	0.11	0.7403
Block 61			

AGCCACA	0.750, 0.530	1.51	0.2191
TATTTTG	0.125, 0.230	0.487	0.4854
AATTTTG	0.125, 0.111	0.014	0.9047
AATTTCA	0.000, 0.061	0.517	0.4721
AGTCTCA	0.000, 0.054	0.456	0.4994
Block 62			
GAC	0.750, 0.814	0.211	0.6462
TGA	0.125, 0.155	0.055	0.8144
GGC	0.125, 0.020	3.798	0.0513
Block 63			
CCCGGCTA	0.625, 0.471	0.743	0.3886
GTCCGTGT	0.250, 0.230	0.018	0.8931
GCCGGCTA	0.125, 0.191	0.224	0.6363
CCTGCCTA	0.000, 0.081	0.704	0.4014
CCCGGTTA	0.000, 0.017	0.137	0.7109
Block 64			
GACGAATAA	0.500, 0.495	0.001	0.9779
TGCATGCTT	0.375, 0.402	0.024	0.8773
GATAAGCTA	0.125, 0.066	0.434	0.51
GACGAATAT	0.000, 0.012	0.094	0.7597
TGCATGCTA	0.000, 0.010	0.079	0.7782
Block 65			
CACCGTTGT	0.625, 0.510	0.411	0.5213
CCCCGTTGT	0.375, 0.419	0.062	0.8037
TAGTACCAA	0.000, 0.054	0.456	0.4993

Block 66			
TG	1.000, 0.892	0.967	0.3255
AA	0.000, 0.108	0.967	0.3255
Block 67			
CCTCGCT	0.375, 0.503	0.514	0.4736
TGGATCT	0.250, 0.243	0.002	0.965
TGGATTG	0.375, 0.233	0.868	0.3516
TGGATCG	0.000, 0.020	0.165	0.6842
Block 68			
TGGTGACGG	0.750, 0.578	0.951	0.3295
TGGTGACGT	0.000, 0.128	1.174	0.2786
TGGTGGAAT	0.125, 0.098	0.064	0.8003
CAACAGCGT	0.000, 0.074	0.641	0.4234
TGGTGGCGT	0.000, 0.057	0.487	0.4854
TGATGACGT	0.125, 0.047	1.003	0.3167
CAACGACGT	0.000, 0.014	0.11	0.7407
Block 69			
GG	0.875, 0.814	0.192	0.6615
AA	0.125, 0.186	0.192	0.6615
Block 70			
TTCAGAT	0.625, 0.581	0.062	0.8037
CCTGAAG	0.125, 0.172	0.123	0.7259
TTCAAAT	0.125, 0.095	0.083	0.7727
CTCAAAG	0.000, 0.084	0.736	0.3909
TTCAGTT	0.125, 0.061	0.548	0.4592

Block 71			
AGCTCTAGCTCAAGTAACACCCCGCTAGGTACTGTTT	0.625, 0.507	0.436	0.5092
GAGTCCCATCAGATACAAGTCTTATCCCACGTGCCCC	0.125, 0.128	0.001	0.9775
AGCCCTAGCCCAGGTAGCACCCTGCTAGGTACTGTTT	0.125, 0.098	0.064	0.8003
AGCTCTAGCCCAGGTAGCACCCTGCTAGGTACTGTTT	0.000, 0.071	0.61	0.4349
AGCTTTAGCCCAAGTAACACTTTGCTAGGTACTGTTT	0.125, 0.061	0.548	0.4592
AGCTCTAGCCCAAGTAACACCTTGCTAGGTACTGTTT	0.000, 0.061	0.517	0.4721
AGCTCCAGCCCAGGACACGCCTTGCTAGGTACTGTTT	0.000, 0.047	0.397	0.5288
Block 72			
CA	0.750, 0.605	0.69	0.4061
TT	0.250, 0.324	0.197	0.6572
CT	0.000, 0.071	0.61	0.4349
Block 73			
TATTAAGAGGTTCCCCGTTATGGAACCCACGTCACCCGGGGACACGGGGTTT	0.250, 0.182	0.237	0.6266
TGTAAAGACGCTGCCCGGTGTGACACTCCCGTCACCCGGGGACACGGGGTTT	0.000, 0.152	1.428	0.2322
CGTTAAGAGGTTGCTCATTATGAACCTCCCGTCACCAGTGAATGCTACATCG	0.250, 0.135	0.863	0.3529
TATTAAAAGGTCCCCCGTTATAGAACCCACGTCACCCGGGGACACGGGGTTT	0.000, 0.128	1.174	0.2786
TGTTAAGCGGTTGTCAGTCAAGAAACTTCGACTTTTCAGAGGCATGGGGCTT	0.250, 0.091	2.276	0.1314
TGGTAAGAGGTTGCCCGTTATGAAATTCCCGTCACCCGGGGACACGGGGTTT	0.125, 0.077	0.244	0.6216
TATTCCGAGCTTCCCCGTTATGGAACCCACGTCACCCGGGGACACGGGGTTT	0.000, 0.068	0.579	0.4469
TGTTAAGAGGTTGTCAGTCAAGAAACTTCGACTATTCAGAGGCATGGGGTTT	0.000, 0.051	0.429	0.5124
TGTTAAGAGGTTGCCAGTTATGAAACTTCCACTATTCAGGGGCATGGGGTTT	0.000, 0.044	0.367	0.5446
TGTTAAGAGGTTGCCCGTTATGAAACTCCCGTCACCCGGGGGCACGGGGTTT	0.000, 0.024	0.194	0.6599
TGTTAAGCGGTTGTCAGTCAAGAAACTTCGACTATTCAGAGGCATGGGGTTT	0.000, 0.023	0.191	0.662
Block 74			

GGTCGGACGGGGGCGAAGTCCCACGCG	0.875, 0.713	1.01	0.315
AACGTAGTACAGAGAGGACTCCACGCG	0.000, 0.061	0.517	0.4721
AACGTAGTACAAAGAGGACTTGGGATA	0.000, 0.061	0.517	0.4721
AACGTAGTACAAAGAGGACTTGGGGTA	0.125, 0.044	1.166	0.2803
AACGTAGTACAAAGAGGACTCCACGCG	0.000, 0.041	0.338	0.5612
AACGTAGCGGGGACGGGATCCCACGCG	0.000, 0.037	0.308	0.5786
GGCGTAGCGGGGACGGGATCCGACGCG	0.000, 0.020	0.165	0.6842
Block 75			
CATGGTTGTGGGCCT	0.875, 0.770	0.487	0.4853
AACGACGACAAGGCC	0.125, 0.105	0.034	0.8537
CTCTACTGCGAACTT	0.000, 0.041	0.338	0.5612
ATCTACTGCGAACTT	0.000, 0.037	0.308	0.5786
AACGACGGCGAGGCT	0.000, 0.037	0.308	0.5786
Block 76			
AG	0.750, 0.804	0.144	0.7047
CT	0.250, 0.186	0.211	0.6462
Block 77			
CTCATGGG	0.625, 0.632	0.002	0.9687
TGGGCAAA	0.250, 0.226	0.025	0.8749
CGCATGGG	0.125, 0.115	0.008	0.9294
TGGGCAAG	0.000, 0.014	0.109	0.7416
Block 78			
TGGCGCGTGCATGTTTAGGTGATAC	0.625, 0.655	0.032	0.8584
CCATCTACTTGCACCCGATAAGGGA	0.250, 0.250	0	1
CCATCTACTTGTACCCGATAAGGGA	0.125, 0.054	0.743	0.3888

CCATCTATTTGCACCCGATAAGGGA	0.000, 0.034	0.279	0.5971
Block 79			
CGC	0.375, 0.476	0.321	0.571
CAC	0.375, 0.348	0.025	0.8742
GGG	0.125, 0.149	0.035	0.8526
CGG	0.125, 0.027	2.603	0.1067
Block 80			
ACG	0.375, 0.470	0.28	0.5967
GTA	0.375, 0.338	0.048	0.8265
GCG	0.250, 0.182	0.237	0.6266
Block 81			
AGTGTATTCTTAACGTTTGGCG	0.369, 0.385	0.008	0.9283
AACGCGTTCTTAACGTTTGGCG	0.266, 0.281	0.008	0.9278
GACACGTTCTTAACGTTTGGCA	0.115, 0.125	0.006	0.9363
AACGCGACTGGGTTCCAAATTG	0.234, 0.098	1.587	0.2078
AACGCGACTGGATTCCAAATTG	0.000, 0.041	0.338	0.5612
GACACGTTCTTAACGTTTGGCG	0.000, 0.031	0.257	0.6125
Block 82			
GG	0.750, 0.824	0.295	0.5873
AC	0.250, 0.159	0.479	0.4887
AG	0.000, 0.017	0.137	0.7109
Block 83			
GGCG	0.375, 0.323	0.095	0.7583
AGTG	0.250, 0.291	0.063	0.8014
GCCC	0.125, 0.188	0.208	0.6487

GGCC	0.125, 0.153	0.045	0.8317
GGTG	0.125, 0.034	1.833	0.1758
Block 84			
GTCACCCC	0.500, 0.453	0.07	0.7909
ACGGTGGC	0.375, 0.270	0.43	0.5118
ACGGTGGT	0.000, 0.257	2.739	0.0979
Block 85			
TTTCAAGGTCCAACTTCTGCGTCAATTGAGTTGCGTAT	0.000, 0.189	1.859	0.1728
TTTCAAGGTCCAACGAGTGCGTAAATCACGTTGCGTGT	0.125, 0.159	0.067	0.7957
TCATGAACCTCTGTTAGTCGACAGAATGAGCCATGTGT	0.125, 0.139	0.012	0.9127
TTATGAACCTCTGTTAGTCGACAGAATGAGCCGTGTGT	0.250, 0.128	1.008	0.3155
CTTTGGACCCTTGTTAGACGGTAACATGAGTCGTACGC	0.000, 0.091	0.801	0.3708
TTTCAAGGTCCAACGAGTGCGTAAATTGCGTTGCGTGT	0.375, 0.068	10.524	0.0012
TTTCAAGGTCCAACGAGTGCGTAAATCGCCTTGCGTGT	0.125, 0.064	0.468	0.4938
TTTCAAGGTCCAACGTCTGCGTCAATTGAGTTGCGTAT	0.000, 0.061	0.517	0.472
TTTCAAGGTCCAACTACTGCGTCAATTGAGTTGCGTAT	0.000, 0.047	0.395	0.5299
TTTCAAGGTCCAACTTCTGCGTCAATTGAGTTGCGTGT	0.000, 0.013	0.108	0.743
Block 86			
GCATC	0.000, 0.287	3.189	0.0741
CCGAT	0.375, 0.270	0.431	0.5117
GCGTC	0.500, 0.199	4.286	0.0384
GCGAC	0.125, 0.142	0.018	0.8924
GTGTC	0.000, 0.095	0.833	0.3613
Block 87			

CACCTCGCTCGAGGGACCCAGCCGCTCTCCGTTCTACTTTTCCCGACAGGCCGGCGGCGCGGTTATACACATT	0.250, 0.145	0.677	0.4105
TACTCCGCCCGGGATACCCAATTGCGCTATATCTTGCTCGTCCCAGCATTGTAGCGATAAGACCATACCACTT	0.250, 0.128	1.008	0.3153
CACCTCGCTCAAGGTATCCTGCTGCTCACCGTTTTGCCTGTCCCGGTAGTCCGGCGGCGCGATCATACACATT	0.000, 0.135	1.245	0.2646
CACCTCGCTCGAGGTACCCAGCCGCTCTCCGTTCTACTTTTCCCGACAGGCCGGCGGCGCGACCATACACATT	0.125, 0.122	0.001	0.977
CTCCTCGCTTGAGGGACCCAGCCGTTCTCCGTTCTAATTTCTTGACAGGCCGGGGGCGCGGTTATACACATT	0.000, 0.111	1	0.3172
CACCTCGCTCGAGGTACCCAGCCGCTCTCCGTTCTGCTTGTCCCGACAGGCCGGCGGCGCGACCATACACATT	0.125, 0.101	0.048	0.8273
TATTCTATCCGGGATGCTTAGTTGCGTTATACTTTGCTTGCACCAGCCTTGCAACAGCGCAACCGCATCACAC	0.000, 0.088	0.768	0.3807
TACTCCGCCCGGAATACCCAATTACGCTATATCCCGCTCGTCCCAGCATTGTAGCGATAAGACCATGCCACTT	0.125, 0.028	2.514	0.1128
TACTCCGCCCGGAATACCCAATTACGCTATATCTCGCTCGTCCCAGCATTGTAGCGATAAGACCATGCCACTT	0.125, 0.023	3.241	0.0718
CACCTCGCTCAAGGTATCCTGCTGCGCTCCGTTCTACTTGTCCCGACAGTCCGGCGGCGCGATCATACACATT	0.000, 0.020	0.16	0.6894
Block 88			
TCCGGAGTGCC	0.125, 0.384	2.226	0.1357
TCGCGTGTGCT	0.500, 0.280	1.839	0.1751
CTCCATTCGGT	0.125, 0.145	0.026	0.8723
TCCGGTGTGCC	0.000, 0.089	0.778	0.3777
TCCCATTTACC	0.250, 0.054	5.369	0.0205

TCCGGTTTGCT	0.000, 0.017	0.137	0.7109
TCCCATTTGCC	0.000, 0.014	0.11	0.7407
Block 89			
CTGTCTGTCCGCGAAGTCGTA	0.125, 0.375	2.09	0.1482
CTGTTTGTCTGCAAAGTCAAA	0.250, 0.215	0.055	0.8138
TGGCCCATCTAAGGTACCGAG	0.000, 0.128	1.172	0.2789
CTGTCTGTCTGCAAAGCCGAA	0.000, 0.095	0.834	0.3613
TGTCCCAGTTGCGGTGCTGAA	0.250, 0.054	5.369	0.0205
CTGTCTGTCTGCAAAGTCAAA	0.250, 0.048	6.256	0.0124
CTGTCTGTCTGCAAAGTCGAA	0.000, 0.024	0.194	0.6599
TGGCCCATCTAAGATACCGAG	0.125, 0.017	4.663	0.0308
CTGTTTGTCTGCGAAGTCGTA	0.000, 0.014	0.117	0.7319
Block 90			
TTGCGCCCC	0.125, 0.432	3.014	0.0826
TCATATACC	0.250, 0.213	0.064	0.8002
TTACGCCCC	0.000, 0.128	1.169	0.2795
TCATGCCCC	0.000, 0.081	0.699	0.403
GCATGCCTT	0.250, 0.057	4.93	0.0264
TCATGTACC	0.250, 0.051	5.86	0.0155
TTATGCCCC	0.000, 0.021	0.169	0.6812
TCACGCCCC	0.125, 0.017	4.578	0.0324
Block 91			
CAGGTTGGGTTATTATCTCG	0.500, 0.476	0.017	0.8949
TAGGTGCCCTTAACGCATTG	0.125, 0.267	0.809	0.3685
TGTCGGGGCCAGACGCACTC	0.250, 0.182	0.237	0.6266

TAGGGTGGCTTATCATCTTG	0.125, 0.024	3.123	0.0772
TAGGGGCCCTTAACGCATTG	0.000, 0.020	0.165	0.6842
Block 92			
AG	0.625, 0.615	0.003	0.9536
TC	0.250, 0.203	0.107	0.7432
TG	0.125, 0.182	0.173	0.6771
Block 93			
TGTGCC	0.499, 0.466	0.034	0.8536
TGTGAT	0.125, 0.287	1.01	0.315
CGCGAC	0.250, 0.139	0.797	0.3719
CTTTAC	0.000, 0.051	0.425	0.5144
CGTGAC	0.124, 0.027	2.568	0.1091
Block 94			
GC	0.500, 0.486	0.006	0.9399
AA	0.500, 0.422	0.193	0.6608
GA	0.000, 0.091	0.801	0.3708
Block 95			
CGGTTGACCAACCGGGTCATCGTGGGATGTTCCGGCTGGCCGAATAAAGATCATTTCTAA	0.499, 0.439	0.115	0.7347
GAACCTTTTACATACTCTTCAACTTAGCACGTTAATAAATTACGCCGTAGGTGCACGCGG	0.250, 0.163	0.425	0.5143
CAGCTGACCACACGGGTCATCGTTGAATGTTCCGGCTGGCCGAATAAAGATCATTTCTAA	0.000, 0.088	0.773	0.3792
GAGCTGACCACACGGGTCATCGTTGAATGTTCTGGCTGGCCGAATAAAAATCATTCGTGG	0.000, 0.075	0.646	0.4217
CAGCTGACCACCCGGGTCATCGTGGGATGTTCCGGCTGGCCGAATAAAGATCATTTCTAA	0.000, 0.044	0.37	0.5432
GAACTTTTTCCATACGCCTTCGTTGAATGTTCCGGCTGGCCGAATCATAATCACACGTGG	0.000, 0.034	0.281	0.5958
GAACTTTTTCCATACGCCTTCGTTTAGTGTTTTAATAAATTGCATCATAATCACACGTGG	0.000, 0.031	0.252	0.6154
CAGTTGACCAACCGGGTCATCGTGGGATGTTCCGGCTGGCCGAATAAAGATCATTTCTAA	0.000, 0.024	0.195	0.6588

Block 96			
AC	0.750, 0.716	0.044	0.8342
GT	0.250, 0.284	0.044	0.8342
Block 97			
CGGGAATCTTATTATCACGCTCCCGGATTTCAAGAATTGGAATATCGCTGCACC	0.500, 0.574	0.176	0.675
TCAACGCCATATTACCGTGTTAGCGGGTTTCAACATTCGAAATATCCCCGGACA	0.250, 0.135	0.863	0.3529
CCAACGCGTAGCCGCTGCCTCCCGACGCCCTGGCCACCCGTTGGCTGATACGTA	0.000, 0.088	0.768	0.3807
CGAACGCGTAGTCGCCGCGTTCCCGGGTTCTAACAACCGGATGGCTGCTGCACC	0.250, 0.044	7.052	0.0079
CGAACGCGTAGTCGCCGCCTCCCGACGCCCTGGCAACCGGATGGCTGCTACACA	0.000, 0.024	0.194	0.6599
CCAACGCGTAGCCGCCGCCTCCCGACGCCCTGGCCACCCGTTGGCTGATACGTA	0.000, 0.020	0.165	0.6842
CGAACGCGTAGTCGCCGCGTTCCCGGGTTCTAACAACCGGATGGCTGCTGCACA	0.000, 0.017	0.137	0.7109
TCAACGCCTTATTACCGTGTTAGCGGGTTTCAACATTCGAAATATCCCCGGACA	0.000, 0.017	0.137	0.7109
CGGGAATCTTATTATCGCGTTCCCGGGTTTCAACAATTGGAATATCGCTGCACC	0.000, 0.014	0.11	0.7407
CGGGAATCTTATTATCGCGTTCCCGGGTTTCAACAATTGGAATATCGCTGCACA	0.000, 0.014	0.11	0.7407
Block 98			
AGGATAATGGGCCCTACAAGGATTTATCTGCTCTCTGGT	0.375, 0.512	0.584	0.4446
TAAGTGCCTTATTGCGTGAGGACTGAACCCACTCTCCGA	0.250, 0.176	0.294	0.5875
AGGATAATGGGCCCTACAAGGATTTATCTGCTCTCTGGA	0.125, 0.076	0.262	0.6086
AGGATAATGTGCCCTACAAGGATTTAACTGCTCTCTGGT	0.125, 0.071	0.341	0.559
TAAGGGCCTTATTCCGTATTAGTCGGATCGACCCTCGAA	0.125, 0.064	0.468	0.4937
TAAGTGCCTTATTCCGTATTAGTCGGATCGACCCTCGAA	0.000, 0.034	0.28	0.597
TAAGTGCCTTATTCCGTAATGATTGGACCGACCCTCGGA	0.000, 0.024	0.194	0.6599
TAAGTGCCTTATTCCGTGAGGATTGAACCGACTCTCCGA	0.000, 0.020	0.165	0.6842
TAAGTGCCTTATTCCGTAATAGTCGGATCGACCCTCGGT	0.000, 0.012	0.097	0.7558
Block 99			

TTACATAGAGGCACAGTTCTT	0.625, 0.743	0.566	0.4517
CCGTGCCAATATGTGTCGTCA	0.125, 0.115	0.008	0.9296
CCGTGCCAGTATGTGTCGTCA	0.125, 0.074	0.286	0.5927
TTACACCGAGGCACAGTTCTT	0.125, 0.017	4.693	0.0303
CTGTGCCAATATGCGTCGTCA	0.000, 0.020	0.165	0.6842
Block 100			
AATCTT	0.375, 0.500	0.487	0.4853
GGCTCA	0.625, 0.486	0.598	0.4393
Block 101			
GGGATACTAAGGAAACGATGCTAAACA	0.250, 0.318	0.165	0.685
ATCGCTGCGGAAGGCTAGCTTCTGTTG	0.125, 0.206	0.315	0.5744
GTGGTACTAAGGAAACGATGCTAAACA	0.125, 0.196	0.251	0.6166
GTGGCACTAAGGAAACGATGCTAAACA	0.125, 0.152	0.044	0.8333
ATCGCTGCGGAAGGCTAGCTTCAAACG	0.375, 0.105	5.728	0.0167
GTGGCACTGAGGAAACGATGCTAAACA	0.000, 0.014	0.11	0.7407
Block 102			
CT	0.375, 0.652	2.61	0.1062
TA	0.625, 0.345	2.685	0.1013
Block 103			
TTC	1.000, 0.875	1.139	0.286
CGT	0.000, 0.105	0.933	0.3341
CGC	0.000, 0.020	0.165	0.6842
Block 104			
CTTATG	0.250, 0.314	0.149	0.6991
TCCCTA	0.500, 0.304	1.4	0.2367

CTTCCG	0.125, 0.186	0.192	0.6615
CCCCTA	0.000, 0.101	0.9	0.3429
CCTCTA	0.125, 0.095	0.083	0.7727
Block 105			
TGC	0.125, 0.375	2.092	0.148
CTT	0.500, 0.331	0.997	0.3179
CGC	0.375, 0.277	0.372	0.5422
CGT	0.000, 0.014	0.11	0.7403
Block 106			
CC	1.000, 0.730	2.936	0.0866
GG	0.000, 0.243	2.548	0.1104
GC	0.000, 0.020	0.166	0.6833
Block 107			
TAAAAT	0.750, 0.743	0.002	0.9656
CGTGGC	0.250, 0.247	0	0.9826
Block 108			
AATAGTGTCTTACAGCCTTTAAC	0.500, 0.355	0.715	0.3979
GCTGCCGCACCCTGGTGCCCAGG	0.250, 0.209	0.077	0.7814
AACGCTACACCCTGATGCCCCAC	0.125, 0.128	0.001	0.9775
AATGCCACACCCTGGTGCCCAAC	0.000, 0.115	1.035	0.3091
GCTGCCGCACCCTGGTGCCCAAC	0.125, 0.064	0.469	0.4936
AATGCCGTATTACGGTGCCCAAC	0.000, 0.061	0.517	0.4721
AATAGTGTATTACAGCCTTTAAC	0.000, 0.044	0.367	0.5446
AATGCCGCACCCTGGTGCCCAAC	0.000, 0.017	0.137	0.7109
Block 109			

CGGTGAGTAT	0.625, 0.466	0.788	0.3746
TTAAAGAAAC	0.250, 0.209	0.077	0.7814
TTATAGAAAT	0.125, 0.132	0.003	0.9555
TTATAGAAGC	0.000, 0.108	0.967	0.3255
TGATGAATAT	0.000, 0.061	0.517	0.4721
TTATAGAAAC	0.000, 0.024	0.194	0.6599
Block 110			
GG	0.875, 0.807	0.23	0.6313
CC	0.125, 0.193	0.23	0.6313
Block 111			
CGAGGTTCGATTGAAAGGGGTCCTAGAAAATAATAGAATTGGC	0.625, 0.436	1.131	0.2875
TAAGGTTCGATTGGAAGGGGTCCTAAAAAATAGTAGCATTGGC	0.250, 0.216	0.052	0.819
TGTAACGTAGACCACGAAAAGGTCGAGGCTCGGGGACCCCAAT	0.125, 0.132	0.003	0.9555
TAAGGTTCGATCCACGAAAAGGTCGAGGCTCGGGGACCCCAAT	0.000, 0.108	0.967	0.3255
TGAGGTTCGATTGGAAGGGGTCCTAAAAAATAGTAGCATTGGC	0.000, 0.044	0.367	0.5446
CGAGGTTCGATTGAAAGGGGTCCTAGAAAATAGTAGAATTGGC	0.000, 0.027	0.222	0.6376

Table S5. Functional annotation (Haploreg v4.1) of non-coding haplotypes identified as significantly associated with "having 10 or more outpatient surgeries" among Val122Ile carriers.

LD Block	Variant	GERP cons	SiPhy cons	Promoter histone marks	Enhancer histone marks	DNAse	Proteins bound	Motifs changed	NHGRI/EBI GWAS hits	GRASP QTL hits	Selected eQTL hits
#12	rs8098472							p300			
	rs2850275							FAC1,Irf,PU.1,TCF12,p300			
	rs28644250							AFP1,AhR,Dbx1,Sox			
	rs4627446							EWSR1-FLI1,Egr-1,Myc,Pax-4,SP1,STAT,TCF12,UF1H3BETA,WT1,ZNF263,Zfp281,Znf143			
	rs9950324							CDP			
	rs2000806							Irx,XBP-1			

	rs2000805			Foxm1,HNF6	
	rs1943540		ESDR	DBP,Foxa,GR,HDAC2,HNF4,TCF12,p300	
	rs4799557		ESDR	Rad21	
	rs17711466	ESDR	IPSC,BRN,BRN	SP2	2 hits
	rs9962586	ESDR, KID, LNG, BLD	ESDR	ERalpha-a,Sox	
	rs56352067	HRT		SIX5,Zbtb3	
	rs1532283		VAS	BRCA1,HDAC2,Irf,TATA,p300	
#14	rs68134899			Bbx,Foxa,Foxd3,HDAC2,HMG-IY,Hbp1,Pou1f1,Pou3f2,RREB-1,STAT,Sox,Zfp105,p300	
	rs9953253			AP-1,Irf,PRDM1	
	rs113442636			GR,TATA,YY1	

	rs10164247			Nkx3,Sox
	rs111561958			AhR
	rs72918851			AhR,Egr-1,GR,PU.1,SREBP,STAT,TAL1,UF1H3BETA
	rs28546651			Cdx,Foxd3,HDAC2,HMG-IY,Irf,Mef2,Pax-4,Zfp105
	rs16960912			Nkx2
	rs72918852			AIRE,Nkx2,Nkx3,Pou5f1
	rs16948575			Foxp1,Hmx,Sox
	rs28522789			Pou3f3,STAT
	rs3855662		ESC, IPSC	Maf
#15	rs66663800			AP-3,DMRT2,Pou2f2
	rs17798291			CTCF,Egr-1,Ets,FEV,GATA,Pax-5,Rad21

#16	rs72918853					
	rs9957088			BLD		CDP,Foxf1,Foxi1,Foxj2,Foxo,Foxq1,Mrg,NF-Y,Pbx-1
	rs28758452			BLD		Lhx3,Nkx6-1,Pou2f2,Pou3f1,p300
	rs62089046					E2F,Foxj1,Nanog,Pou2f2,Pou3f2,Sox,YY1,Zfp105
	rs8092513					CTCF,Pou2f2
	rs2082771			BRN	KID,MUS	Zbtb3
	rs2420204					Cdx2,Hoxa10,Hoxb9,Hoxc9,Hoxd10,Myf,TAL1
	rs2117612					Foxq1,Gfi1,HNF1,PLZF
	rs2117613					
	rs58982770					SIX5
	rs1602894					

rs10163717					ZBRK1
rs2082773					AIRE,RXRA
rs10163924					Foxj1,Foxp1,Gfi1,RREB-1,YY1
rs12326186					Osr
rs3910339					
rs9962221					
rs9952824					Arid3a,Arid5a,Dbx1,HMG-IY,HNF1,Hlx1,Hoxa10,Hoxa9,Hoxb13,Hoxd10,Hoxd8,Lhx3,Ncx,Nkx6-1,PLZF,Pou3f2,Pou3f4,Pou6f1,Sox,TATA
rs7242206			BRST, BRN		Foxp3,Irf,PLZF,Pou2f2,Sox
rs1865515			BRST, BRN, SKIN	BRN,BRN	AP-1,Irf,TCF4

	rs116454306	SKIN, LNG, PLCNT, BRST	SKIN,BRST,BLD,SKIN	MAFF,MAFK,GATA2,JUND	HDAC2,Irf,Nanog,Pou5f1,Sox	
	rs1658100				CIZ,Dbx2,HNF1,Lhx3,Nkx3,Pou1f1,Pou3f2,Pou3f3,Sox	2 hits
	rs11081678	SKIN			E2A,Myf,Pax-1,Rad21,SMC3,TAL1,ZEB1	1 hit
#23	rs1790696	ESC, BRST, SKIN				3 hits
	rs1658101	ESC, IPSC, BRST, SKIN, LNG			Hoxa5,SIX5	6 hits
	rs74564112	ESC, IPSC, SKIN	LNG		Crx,Dmbx1,EWSR1-FLI1,Otx2,TFII-I,Zfp410	
	rs1595355	ESC, IPSC, SKIN	BRST,LNG,BRST		Foxk1,Rhox11	6 hits

rs1790695	IPSC, LIV	SKIN	RXRA,Zfp187	6 hits
rs114554358	LIV		Cdc5,Nkx2,PLZF	
rs116561824			Foxl1	
rs1595356			CEBPB,Nanog,Pou5f1,SRF	2 hits
rs16961188	BRST	IPSC,BRST	Maf,Nanog,Pax-8,Pou2f2	
